# Recent Advances in Indazole-Containing Derivatives: Synthesis and Biological Perspectives

**DOI:** 10.3390/molecules23112783

**Published:** 2018-10-26

**Authors:** Shu-Guang Zhang, Chao-Gen Liang, Wei-Hua Zhang

**Affiliations:** Jiangsu Key Laboratory of Pesticide Science, College of Sciences, Nanjing Agricultural University, Nanjing 210095, China; shuguangz@njau.edu.cn (S.-G.Z.); 2016111015@njau.edu.cn (C.-G.L.)

**Keywords:** indazole, synthesis, biological activities

## Abstract

Indazole-containing derivatives represent one of the most important heterocycles in drug molecules. Diversely substituted indazole derivatives bear a variety of functional groups and display versatile biological activities; hence, they have gained considerable attention in the field of medicinal chemistry. This review aims to summarize the recent advances in various methods for the synthesis of indazole derivatives. The current developments in the biological activities of indazole-based compounds are also presented.

## 1. Introduction

**Figure 1:**
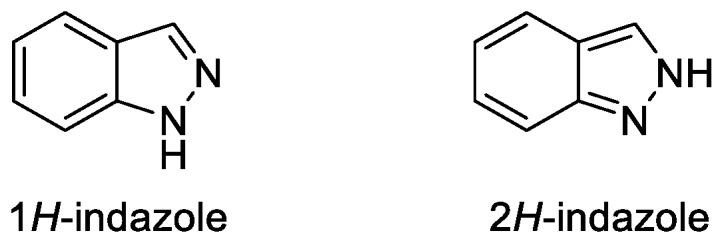
Indazole nucleus.

The nitrogen-containing heterocycles are important building blocks for many bioactive natural products and commercially available drugs. As pharmacologically important scaffolds, they have attracted considerable attention from chemists [1]. Indazoles are one of the most important classes of nitrogen-containing heterocyclic compounds bearing a bicyclic ring structure made up of a pyrazole ring and a benzene ring. Indazole usually contains two tautomeric forms: 1*H*-indazole and 2*H*-indazole (Figure 1). Since 1*H*-indazole is more thermodynamically stable than 2*H*-indazole, it is the predominant tautomer [2].

Indazole derivatives scarcely occur in nature, but this particular nucleus in a variety of synthetic compounds possesses a wide range of pharmacological activities, such as anti-inflammatory, antiarrhythmic, antitumor, antifungal, antibacterial, and anti-HIV activities [3,4,5,6,7,8].

**Figure 2:**
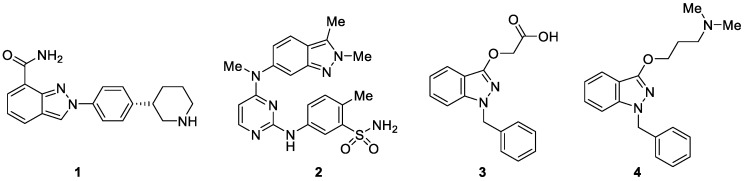
Chemical structure of indazole-containing drugs **1**–**4**.

Diversely substituted indazole-containing compounds furnished with different functional groups represent significant pharmacological activities and serve as structural motifs in drug molecules. For example, niraparib **1** (Figure 2) has been widely used as an anticancer drug for the treatment of recurrent epithelial ovarian, fallopian tube or primary peritoneal, breast and prostate cancer [9]. Pazopanib **2** (Figure 2) is a tyrosine kinase inhibitor, which has been approved by the FDA for renal cell carcinoma [10]. Bendazac **3** and Benzydamine **4** are two commercially available anti-inflammatory drugs, which contain the 1*H*-indazole scaffold (Figure 2) [11].

In light of indazole scaffolds exhibiting a broad spectrum of pharmacological activities, numerous methods have been developed to construct of these heterocycles with better biological activities.

There are some excellent reviews, which have been published on the biological properties of this class of compounds [12,13,14]. This review serves as a comprehensive overview of recent literature that references the synthesis and biological activities of novel indazole-containing derivatives.

## 2. Synthesis Route for Indazole Derivatives

### 2.1. Synthesis of 1H-Indazole

**Scheme 1:**
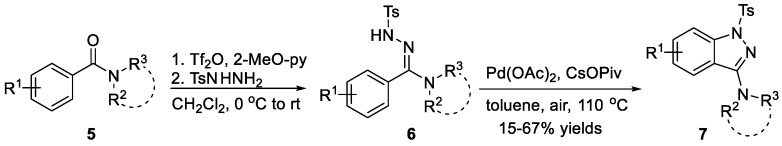
Synthesis of 1*H*-indazoles **7** from tertiary amides **5**.

**Scheme 2:**
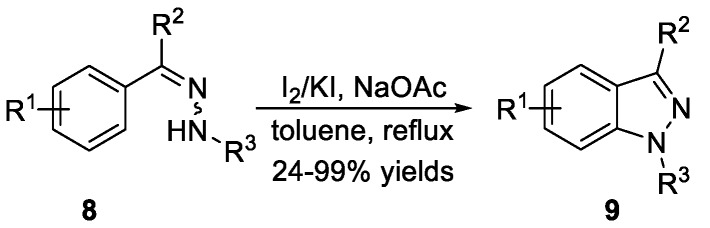
Synthesis of 1*H*-indazoles **9** from hydrazones **8** via iodine-mediated aryl C-H amination.

**Scheme 3:**
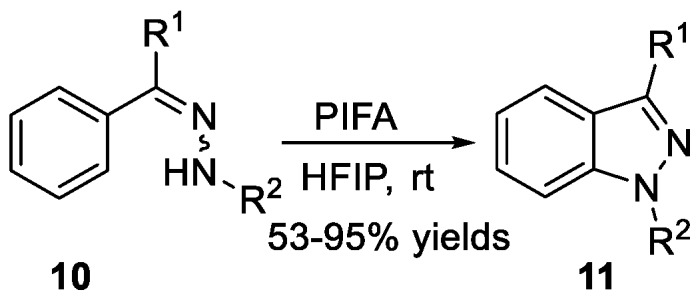
Synthesis of 1*H*-indazoles **11** from hydrazones **10** via PIFA-mediated aryl C-H amination.

The synthesis of 1*H*-indazoles **7** from aminohydrazones **6** with an intramolecular ligand-free palladium-catalyzed C-H amination reaction has been reported by Charette et al. [15]. Substituted aminohydrazones were easily prepared based on trifluoromethanesulfonic anhydride-mediated activation of tertiary amides **5**, and the addition of nucleophilic hydrazides (Scheme 1). Chang et al. [16] treated diaryl and *tert*-butyl aryl ketone hydrazones **8** with iodine in the presence of potassium iodide and sodium acetate obtaining 1*H*-indazoles **9** via direct aryl C-H amination, as shown in Scheme 2. The synthesis of 1*H*-indazoles **11** from arylhydrazones **10** through direct aryl C-H amination using [bis-(trifluoroacetoxy)iodo]benzene (PIFA) as an oxidant was described by Zhang et al. (Scheme 3) [17]. The reaction displayed good functional group compatibility and provided the corresponding compounds in good yields. It is worth noting that both of the two following examples underwent a metal free catalyzed process.

**Scheme 4:**
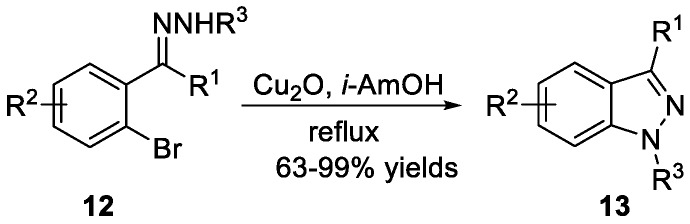
Synthesis of 1*H*-indazoles **13** from *o*-haloaryl *N*-tosylhydrazones **12** catalyzed by Cu_2_O.

**Scheme 5:**
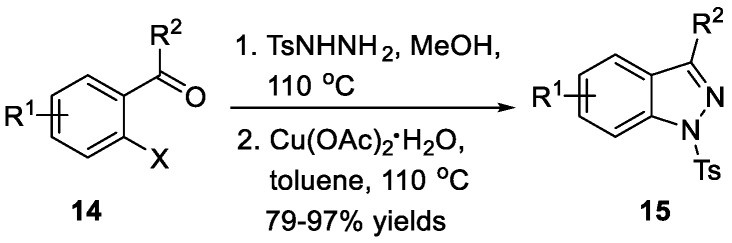
Synthesis of 1*H*-indazoles **15** from *o*-haloaryl *N*-tosylhydrazones **14** catalyzed by Cu(OAc)_2_·H_2_O.

**Scheme 6:**
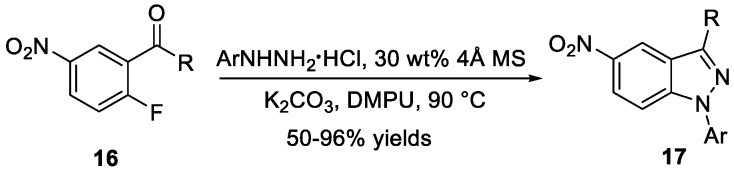
Synthesis of 1-aryl-5-nitro-1*H*-indazoles **17**.

Tang et al. [18] developed a protocol that realized the thermo-induced isomerization of *o*-haloaryl *N*-sulfonylhydrazones and Cu_2_O-mediated cyclization to obtain 1*H*-indazoles **13** (Scheme 4). Moreover, the reaction tolerated various functional groups. Wang et al. [19] disclosed a versatile approach for the synthesis of 1*H*-indazoles **15** via a cyclization of *o*-haloaryl *N*-sulfonylhydrazones **14** using Cu(OAc)_2_·H_2_O as the catalyst, as can be seen in Scheme 5. Compared to Tang’s methods, this approach proceeded at lower temperatures with lower catalyst loading. As an example, Bunce et al. [20] recently explored an efficient route to substituted 1-aryl-1*H*-indazoles **17** (Scheme 6). This transformation involved the preparation of arylhydrazones, followed by deprotonation and nucleophilic aromatic substitution (S_N_Ar) ring closure. Notably, treatment of bromoacetophenone and bromobenzaldehyde with ArNHNH_2_·HCl and 30 wt% of powdered 4 Å molecular sieves in the presence of CuI and K_2_CO_3_ also afforded the desired compounds in good yields.

**Scheme 7:**
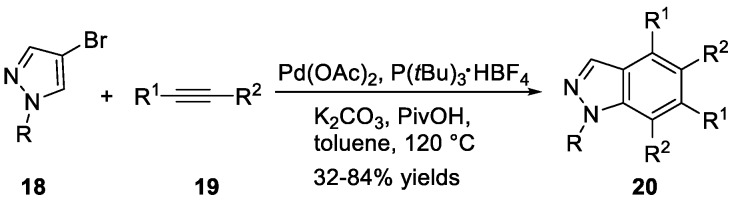
Pd-catalyzed benzannulation of 4-bromopyrazoles **18** for synthesis of 1*H*-indazoles **20**.

**Scheme 8:**
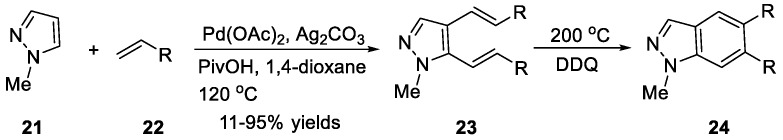
Strategies for dialkenylation of pyrazoles **21** and synthesis of 1*H*-indazoles **24**.

The synthesis of 1*H*-indazoles **20** from pyrazoles **18** and internal alkynes **19** via Pd(OAc)_2_/P(tBu)_3_·HBF_4_ mediated oxidative benzannulation was achieved by Joo et al. (Scheme 7) [21]. Under the optimized conditions, the corresponding 1*H*-indazoles possessing different substituents on the benzene ring were constructed in moderate to good yields. Joo et al. [22] also disclosed another synthesis method for 1*H*-indazoles **24** using a sequence reaction, as shown in Scheme 8. They carried out the regioselective dialkenylation of pyrazoles **21** with electron-deficient alkenes **22** in the presence of Pd(OAc)_2_ and Ag_2_CO_3_. A sequence involving thermal 6π-electrocyclization of dialkenyl pyrazoles and oxidation afforded the desired products.

**Scheme 9:**
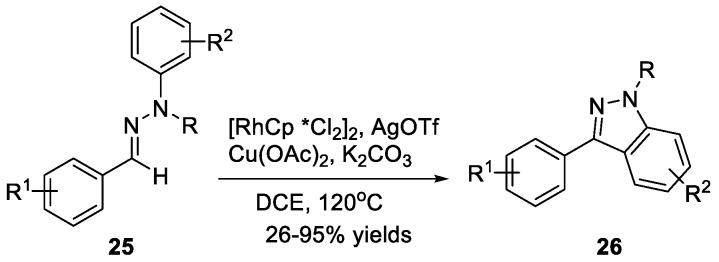
Synthesis of 1*H*-indazoles **26** from aldehyde hydrazones **25**.

Zhu et al. [23] present a simple and efficient strategy to afford 1*H*-indazoles **26** from readily available aldehyde phenylhydrazones **25** through Rh(III)-promoted double C-H activation and C-H/C-H cross coupling (Scheme 9). The reaction tolerated a range of functional groups and lead to the corresponding products in moderate to good yields. 

**Scheme 10:**
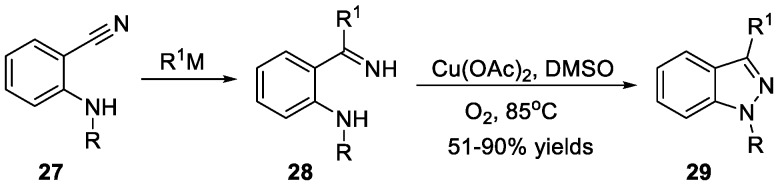
Synthesis of 1*H*-indazoles **29** from 2-aminobenzonitriles **27**.

An efficient and general method to build up 1*H*-indazoles **29** by Cu(OAc)_2_-mediated N-N bond formation using oxygen as the sole oxidant was reported by Chen et al. [24] (Scheme 10). Ketimine species as the key intermediates for this reaction were easily prepared from *o*-aminobenzonitriles **27** and organometallic reagents. The cyclization of ketamine **28** performed by using Cu(OAc)_2_ in the presence of oxygen efficiently converted the desired products in good to excellent yields.

**Scheme 11:**
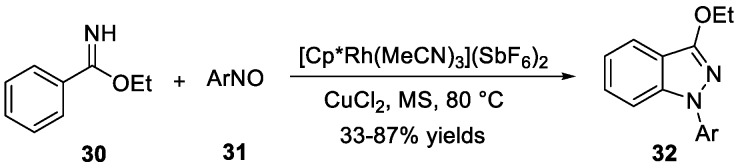
Synthesis of 1*H*-indazoles **32** from nitrosobenzenes **31**.

**Scheme 12:**
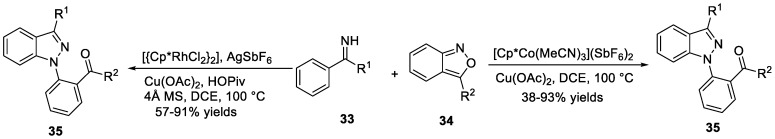
Synthesis of 1*H*-indazoles **35** via Co(III)/Cu(II)-catalyzed C-H activation.

Li et al. [25] accomplished a rhodium and copper catalyzed C-H activation and C-N/N-N coupling of imidate esters or NH imines with nitrosobenzenes **31** to 1*H*-indazoles **32** (Scheme 11). In this transformation, the desired products were obtained under redox-neutral conditions in good yields. In the same year, they [26] observed that 1*H*-indazoles can be synthesized via rhodium-catalyzed amination using anthranil as an aminating reagent under both redox-neutral and oxidative conditions (Scheme 12). With this method, a series of bifunctional products were prepared in good to excellent yields. Both of these two examples involved expensive rhodium catalyst. To address this issue, they [27] explored a synergistic cobalt and copper catalytic system using a similar process to give 1*H*-indazoles **35** (Scheme 12). In this work, anthranil served as both an aminating reagent and organic oxidant. The reactions tolerated various functional groups and good yields were observed.

**Scheme 13:**
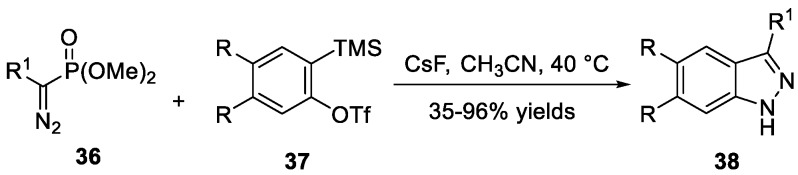
Synthesis of 1*H*-indazoles **38** from α-diazomethylphosphonates **36**.

Peng et al. [28] developed a strategy to access 3-aryl/alkyl-1*H*-indazoles **38** by a 1,3-dipolar cycloaddition reaction of α-diazomethylphosphonates **36** with *o*-(trimethylsilyl)phenyl triflate **37** in the presence of CsF (Scheme 13). In addition, this transformation provided moderate to excellent yields.

### 2.2. Synthesis of 2H-Indazole

**Scheme 14:**
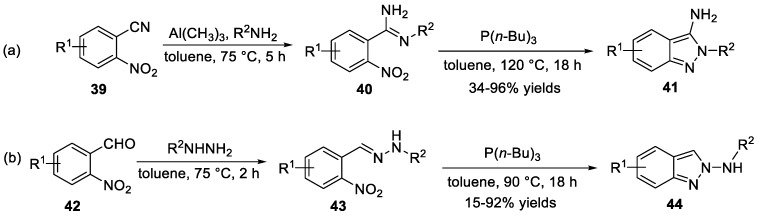
(**a**) Synthesis of 2*H*-indazoles **41** from benzamidines **40** via organophosphorus-mediated N-N bond formation (**b**) Synthesis of 2*H*-indazoles **44** from hydrazones **43** via organophosphorus-mediated N-N bond formation.

Nazaré et al. [29] demonstrated an organophosphorus-mediated reductive cyclization and a subsequent N-N bond formation of substituted benzamidines **40** to construct 3-amino-2*H*-indazoles **41** (Scheme 14a). The substituted benzamidines **40** were synthesized from 2-nitrobenzonitriles **39** through a two-step sequence involving a trimethylaluminium-mediated conversion. The reaction showed good functional group tolerance and satisfactory yields could be obtained. Later, the same group [30] reported a one-pot, two-step approach to generate 2*H*-indazol-2-amines **44** via a reductive cyclization of substituted hydrazones precursors **43** in the presence of organophosphorus reagent (Scheme 14b). In this process, substituted hydrazones **43** were easily prepared from 2-nitrobenzaldehyde **42** and phenylhydrazine.

**Scheme 15:**
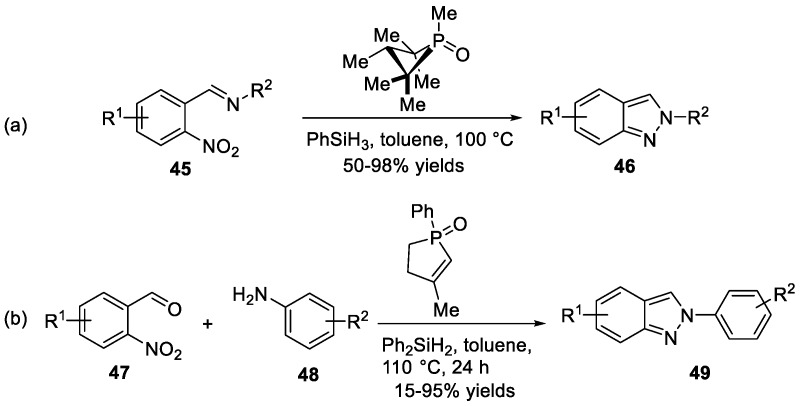
(**a**) Synthesis of 2*H*-indazoles **46** from *o*-nitrobenzaldimines **45** via 1,2,2,3,4,4-hexamethylphosphetane-mediated N-N bond formation (**b**) Synthesis of 2*H*-indazoles **46** from 2-nitrobenzaldehydes **47** and amines **48** by phospholene oxide with organosilanes-mediated N-N bond formation.

**Scheme 16:**
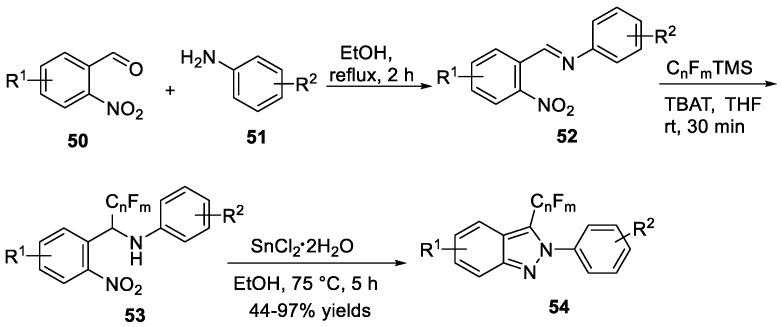
Synthesis of 3-perfluoroalkylated-2*H*-indazoles **54**.

Radosevich et al. [31] developed a useful method to construct 2*H*-indazoles **46** from *o*-nitrobenzaldimines **45** improved by 1,2,2,3,4,4-hexamethylphosphetane (Scheme 15a). In their work, *o*-nitrobenzaldimines **45** underwent deoxygenative N-N bond forming cadogan heterocyclization to provide desired products **46** in the presence of 1,2,2,3,4,4-hexamethylphosphetane and hydrosilane terminal reductant. This approach showed good functional group compatibility. The proposed mechanism revealed that the reaction proceeded via catalytic P^III^/P^IV^=O redox cycling. According to the DFT calculation, (3 + 1) cheletropic addition between the phosphetane catalyst and nitroarene substrate was a turnover-limiting step. Recently, Nazaré et al. [32] reported a one-pot, two-step method to prepare 2*H*-indazoles **49** from 2-nitrobenzaldehydes **47** and amines **48** by domino imine condensation/reductive cyclization in the presence of organophosphorus reagent (Scheme 15b). Similar to Radosevich’s prior work, the organophosphorus agents generated in situ via a redox process from phospholene oxide with organosilanes. The protocol was carried out at 110 °C, tolerating various carboxylic acids, boronic esters, protected amines, phenols to give cyclization products in good yields. In the same year, Nazaré et al. [33] discovered a SnCl_2_·H_2_O mediated reductive cyclization to afford 3-perfluoroalkylated-2*H*-indazoles **54** from α-prefluoroalkylated benzylamine **53** (Scheme 16). This protocol involved the preparation of precursors **52** from 2-nitrobenzaldehydes **50** and anilines **51**, followed by reductive cyclization.

**Scheme 17:**
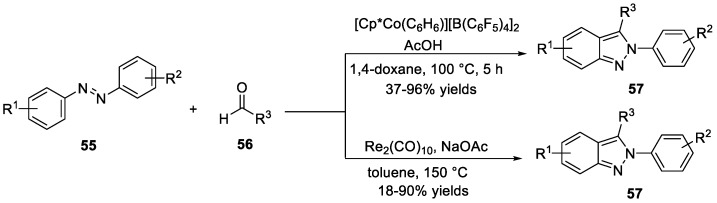
Co(III) or Re-catalyzed synthesis of *N*-aryl-2*H*-indazoles **57** from azobenzenes **55** and aldehydes **56**.

Ellman et al. [34] reported a practical bench-top reaction for the synthesis of *N*-aryl-2*H*-indazoles **57** through Cp*Co(III)-promoted C-H bond functionalization/addition/cyclization cascades (Scheme 17). In this work, treatment of azobenzenes **55** and aldehydes **56** with [Cp*Co(C_6_H_6_)][B(C_6_F_5_)_4_]_2_ and AcOH in 1,4-dioxane at 100 °C led to the corresponding products with moderate to excellent yields. This reaction tolerated a wide range of functional groups and was successfully carried out on a large scale. A similar approach was used by Wang et al. [35] for the synthesis of 2*H*-indazoles **57** via a re-catalyzed C-H transformation of azobenzenes **55** and aldehydes **56** (Scheme 17). It should be mentioned that this reaction is a good example of using isolabe cyclic Re^I^-complex as the catalyst. Remarkably, mechanistic studies disclosed that the aldehyde-insertion step was irreversible in the catalytic cycle.

**Scheme 18:**
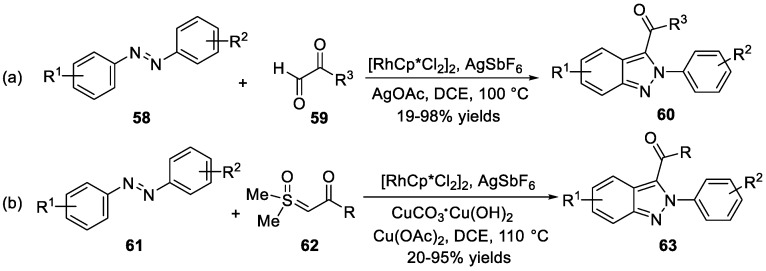
(**a**) Rh-catalyzed synthesis of 2*H*-indazoles **60** from azobenzenes **58** and α-keto aldehydes **59** (**b**) Rh-catalyzed synthesis of 2*H*-indazoles **60** from azobenzenes **61** and sulfoxonium ylides **62**.

The cyclization of azobenzenes **58** with α-keto aldehydes **59** was also realized by Kim et al. [36] using rhodium (III) catalyst (Scheme 18a). This process provided an efficient method to synthesize 3-acylated-2*H*-indazoles **60** via functionalization of C (sp^2^)-H bond in moderate to high yields. A tandem C-H activation/intramolecular annulation reaction between azobenzenes **61** with sulfoxonium ylides **62** for the synthesis 3-acylated-2*H*-indazoles **63** was reported by the same group (Scheme 18b) [37]. In contrast to the reaction conditions in the previous work, Cu(OAc)_2_ and CuCO_3_·Cu(OH)_2_ were utilized instead of AgOAc. In this process, the target products were synthesized with a wide range of substrate scope in good yields.

**Scheme 19:**
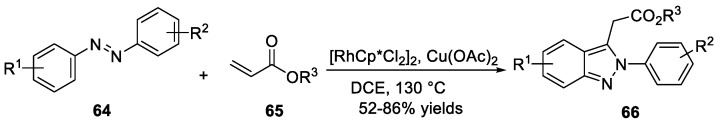
Rh-catalyzed synthesis of 2*H*-indazoles **66** from azobenzenes **64** and acrylates **65**.

Xi et al. [38] reported rhodium (III)-catalyzed C-H functionalization of azobenzenes **64** with acrylates **65** to furnish the corresponding 2*H*-indazoles **66** in the presence of Cu(OAc)_2_ with good functional group tolerance and satisfying yields (Scheme 19). Notably, copper acetate played an important role for β-hydride elimination during the course of transformation.

**Scheme 20:**
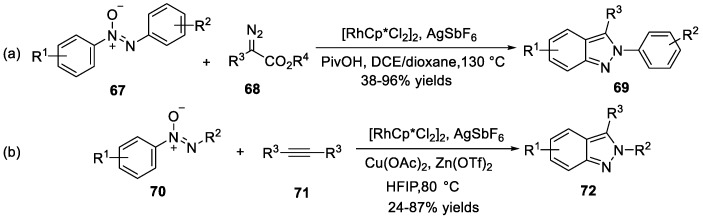
(**a**) Rh-catalyzed synthesis of 2*H*-indazoles **69** from azoxybenzenes **67** and diazoesters **68** (**b**) Rh-catalyzed synthesis of 2*H*-indazoles **72** from azoxy **70** and alkynes **71**.

You et al. [39] discovered a rhodium (III)-catalyzed tandem C-H alkylation/intramolecular decarboxylative cyclization that used azoxy compounds **67** and diazoesters **68** for the synthesis of 3-acyl-2*H*-indazoles **69** (Scheme 20a). In this reaction, azoxy compounds **67** reacted with diazoesters **68** under the catalysis of [Cp*RhCl_2_]_2_ in the presence of AgSbF_6_ to furnish the corresponding 2 *H*-indazoles products with a broad functional group tolerance. Another example regarding the application of similar strategy for the synthesis of 2*H*-indazoles **72** was disclosed by the same group (Scheme 20b) [40]. Under the modified catalytic system, azoxy compounds **70** and alkynes **71** underwent C-H activation/cyclization cascades to provide 2*H*-indazoles **72** in good yields.

**Scheme 21:**
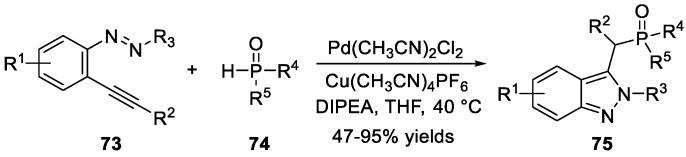
Cu/Pd-catalyzed synthesis of 2*H*-indazoles **75** from 2-alkynyl-azobenzenes **73**.

Lin et al. [41] introduced a Cu/Pd cooperatively catalyzed tandem C-N and C-P bonds formation reaction of 2-alkynyl azobenzenes **73** and compounds **74** for the construction of 2*H*-indazoles **75** (Scheme 21). In this approach, the phosphorated 2*H*-indazoles were synthesized in moderate to good yields, displaying good functional groups tolerance.

**Scheme 22:**
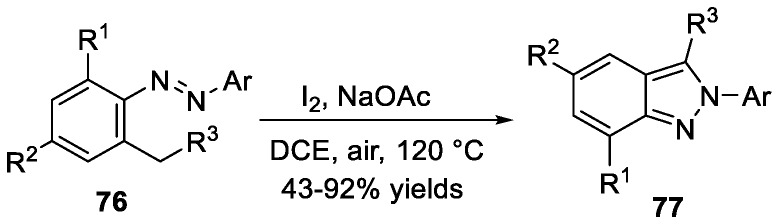
I_2_-mediated synthesis of 2*H*-indazoles **77** from *ortho*-alkylazobenzenes **76**.

Xi et al. [42] developed an efficient iodine-mediated synthesis of 2*H*-indazoles **77** from *ortho*-alkylazobenzenes **76** via benzyl C-H functionalization (Scheme 22). With this method, a variety of 2*H*-indazoles bearing various functional groups were prepared in moderate to good yields. Mechanism studies suggested iodine assisted hydrogen transfer from the benzylic position to nitrogen. DFT calculations revealed that it was a radical chain mechanism. Subsequently, the same group [43] expanded the scope of the substrates. Under similar reaction conditions, using CuI as oxidation promotor and oxygen as a terminal oxidant, *ortho*-alkylazobenzenes could be converted to corresponding products via C-H bond functionalization with high yields.

**Scheme 23:**
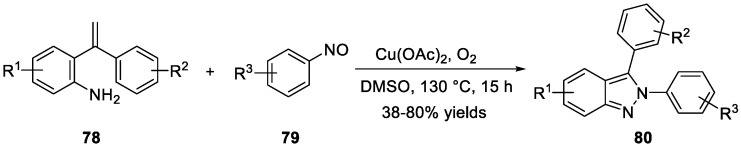
Copper-mediated synthesis of 2*H*-indazoles **80**.

Cheng et al. [44] disclosed a copper-mediated method for the annulation of 2-(1-substituted vinyl) anilines **79** with aryl nitrosos **80** for the synthesis of 2,3-aryl-2*H*-indazoles **80** (Scheme 23). The reaction took place at 130 °C with Cu(OAc)_2_ as a catalyst, O_2_ as an oxidant in DMSO, affording the desired products.

## 3. Biological Applications of Indazole Derivatives

### 3.1. Antitumor Activity

**Figure 3:**
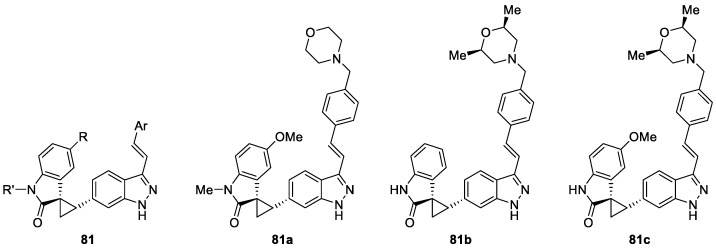
Chemical structures of (1R,2S)-2-(1*H*-Indazol-6-yl)spiro[cyclopropane-1,3’-indolin]-2’-one derivatives **81a**–**c**.

Paul et al. [45,46] reported the synthesis of a series of (1R,2S)-2-(1*H*-indazol-6-yl)spiro [cyclopropane-1,3′-indolin]-2′-one derivatives based on previous bioisosteric scaffold and evaluated their possible potential against Polo-like kinase 4 (PLK4) (Figure 3). As a result, compounds **81a**, **81b**, and **81c** were single-digit nanomolar PLK4 inhibitors. In particular, compound **81c** (CFI-400945) was an effective inhibitor of HCT116 tumor growth in a mouse model of colon cancer and was identified as a novel clinical candidate for cancer therapy.

**Figure 4:**
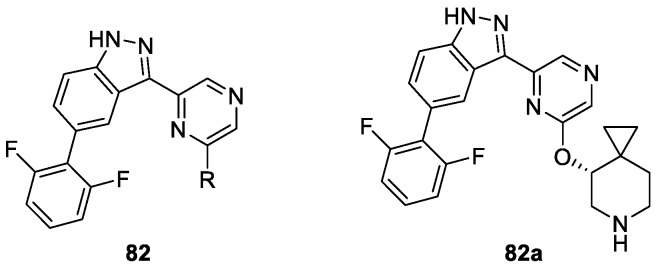
Chemical structures of 5-(2,6-difluorophenyl)-3-(pyrazin-2-yl)-1*H*-indazoles derivatives **82** and **82a**.

Wang et al. [47] described the synthesis of novel 1*H*-indazole derivatives based on systematic optimization of both the piperidine and 2,6-difluorophenyl moieties of 3-(pyrazin-2-yl)-1*H*-indazole as potential inhibitors of pan-Pim kinases (Figure 4). The vitro assay study revealed that among the synthesized and tested compounds, **82a** possessed the strongest activity against Pim-1, Pim-2 and Pim-3 with IC_50_ values of 0.4, 1.1 and 0.4 nM, respectively. Meanwhile, compound **82a** showed moderate cellular potency in KMS-12 BM cell assays with an IC_50_ value of 1400 nM.

**Figure 5:**
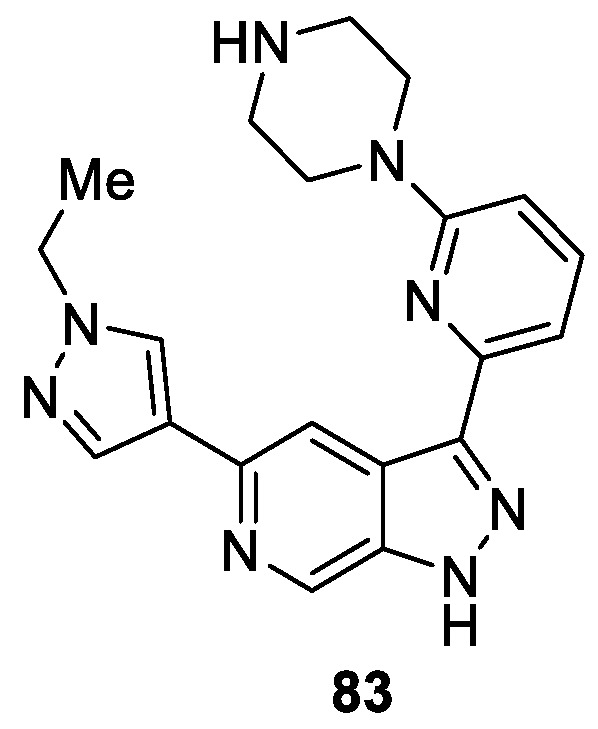
Chemical structure of 1*H*-pyrazolo[3,4-c]pyridine (6-azaindazole) derivative **83**.

Kolesnikov et al. [48] presented the synthesis and evaluation of anti-tumor activity of new 6-azaindazole derivatives. According to the fragment-based approach, they optimized 6-azaindazoles as desirable cores with picomolar biochemical potency against all three Pim kinases, resulting in good cellular potency. Among the synthesized derivatives, compound **83** was the most balanced profile in terms of potency and stability in vivo and was one of the lead compounds in the series. They also found that compound **83** possessed potent antiproliferative activity against the MM1.S multiple myeloma cell line with IC_50_ value of 0.64 μM (Figure 5).

**Figure 6:**
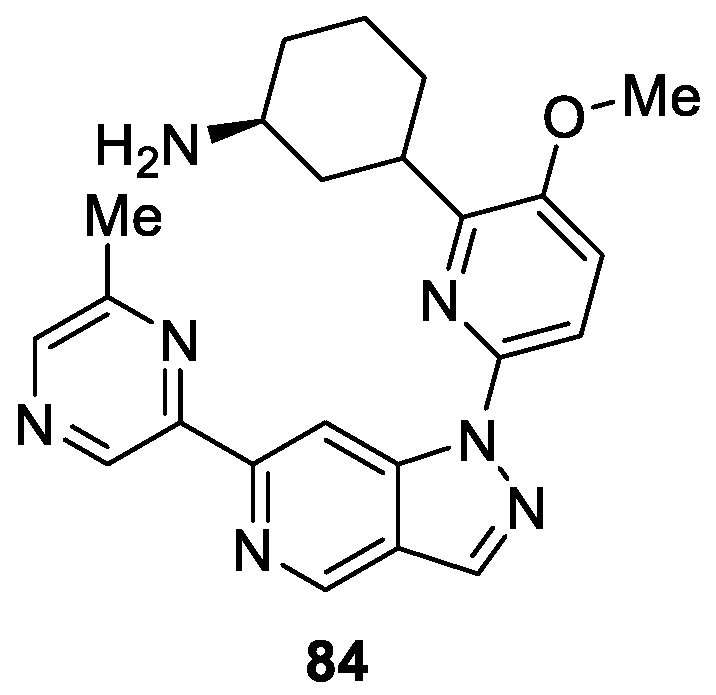
Chemical structure of azaindazole derivative **84**.

Wang et al. [49] designed and synthesized a novel series of azaindazole derivatives, which were tested as inhibitors of pan-Pim Kinases. It was found that compounds **84** possessed the strongest activity against Pim-1, Pim-2 and Pim-3 with *K*_i_ values of 0.03, 0.11 and 0.02 nM, respectively (Figure 6). In particular, **84** was relatively impotent in a two-point hERG patch clamp assay, exhibiting moderate 37% Pim inhibition at 1 μM and 49% Pim inhibition at 10 μM.

**Figure 7:**
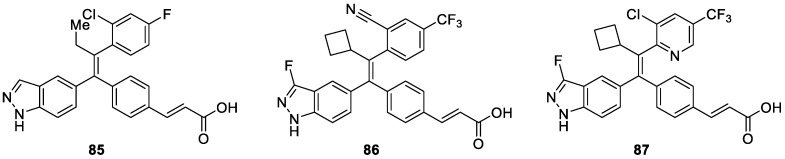
Chemical structures of 1*H*-indazoles derivatives **85**, **86** and **87**.

Indazole-based series of selective estrogen receptor degraders (SERDs) were reported by Govek et al. [50]. A structure-activity relationship study of synthesized derivatives based on compound **85** (Figure 7) revealed that when replacing the ethyl with cyclobutyl group, the corresponding analogs displayed enhanced potency. However, incorporating larger substituents on *para*--position of the upper aryl ring, such as CF_3_, the corresponding compounds showed improved degradation efficacy. They also discovered that by tempering the polarity of lipophilic acids and incorporation of the 3-fluoroindazole motifs, oral bioavailability could be increased. Ultimately, compounds **86** and **87**, which were identified by exploration of ER degradation and antagonism in vitro followed by in vivo antagonism and culminating in oral exposure, could induce tumor regression in a tamoxifen-resistant breast cancer xenograft.

**Figure 8:**
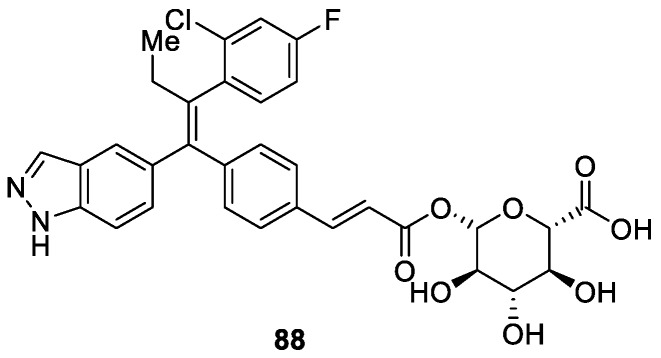
Chemical structure of 1*H*-indazole derivative **88**.

The same year, Smith et al. [51] identified a series of 1*H*-indazole derivatives as orally bioavailable estrogen receptor degraders (SERDs) based on SAR studies on a triphenylalkene scaffold (Figure 8). Among the optimized compounds, **88** was the most efficient degrader of the ER-α with an IC_50_ value of 0.7 nM (Figure 8). Furthermore, **88** exhibited good bioavailability across species and displayed robust activity in tamoxifen-sensitive and tamoxifen-resistant xenograft models of breast cancer.

**Figure 9:**
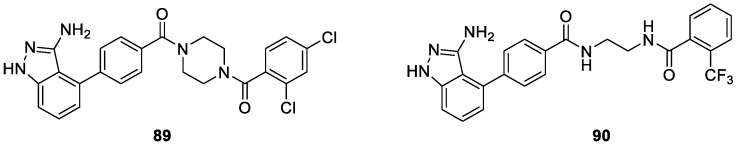
Chemical structures of 1*H*-indazol-3-amine derivatives **89** and **90**.

Wang et al. [52] synthesized a series of *1H-indazol-3-amine* derivatives and evaluated their activity against Bcr-Abl wild type as well as T315I mutant. Among these compounds, **89** and **90** appeared to be the most potent Bcr-Abl inhibitors (Figure 9). In particular, compound **89** served as a promising inhibitor, which exhibited comparable potency with that of Imatinib and inhibited Bcr-Abl^WT^, Bcr-Abl^T315I^ and K562 leukemia cancer cells with IC_50_ values of 0.014, 0.45 and 6.50 μM, respectively. The docking studies indicated that compound **89** bound to Bcr-Abl^WT^ in a similar manner as imatinib.

**Figure 10:**
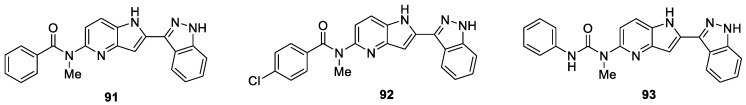
Chemical structures of 3-(pyrrolopyridin-2-yl)indazole derivatives **91**, **92** and **93**.

A novel series of 3-(pyrrolopyridin-2-yl)indazole derivatives were synthesized and evaluated for their anti-proliferative effects on five human cancer cell lines (HL60, KB, SMMC-7721, HCT116, and A549) by Hu et al. [53]. The study revealed that all of the synthesized compounds exhibited vigorous potency against HL60 cell line with IC_50_ values ranging from singe digital nanomolar to micromolar level. Among them, compound **93** was identified to be the most potent inhibitor, with IC_50_ values of 8.3 nM and 1.3 nM against HL60 and HCT116 cell lines, respectively (Figure 10). Furthermore, results of the inhibitory activities against a panel of kinases demonstrated that compounds **91**, **92** and **93** could selectivity inhibit CHK1, CDK2, MEK1, GSK3β, BRAF, IKKβ and PKC. 

**Figure 11:**
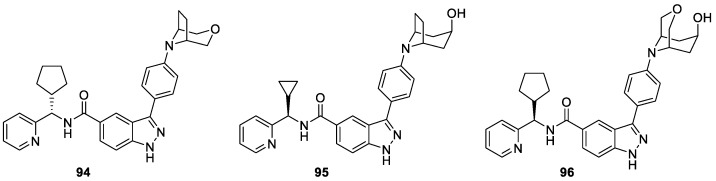
Chemical structures of 3-(4-(heterocyclyl)phenyl)-1*H*-indazole-5-carboxamides derivatives **94**, **95** and **96**.

Pauls et al. [54] synthesized a new set of 3-(4-(heterocyclyl)phenyl)-1*H*-indazole-5-carboxamides derivatives, which were tested as inhibitors of tyrosine threonine kinase (TTK). It was found that compounds **94**, **95** (designated CFI-401870) and **96** were single-digit nanomolar TTK inhibitors, with acceptable off-target activity and good oral bioavailability in rodents (Figure 11). In particular, compound **95** served as the most efficacious of the shortlisted compounds in an HCT116 tumor xenograft model, which also could inhibit the growth of a broad panel of human cancer cell lines including breast, ovarian, colon, prostate, lung and melanoma cell lines. 

**Figure 12:**
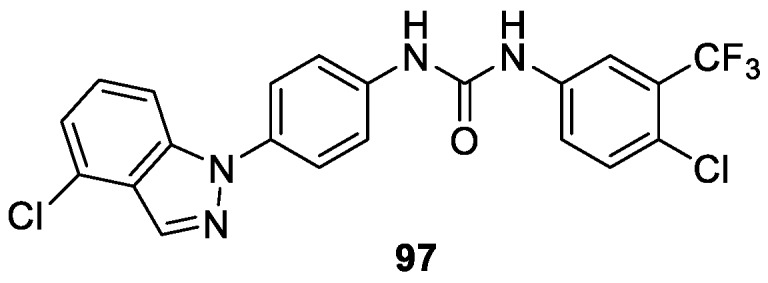
Chemical structure of *N*-(4-(1-(4-chlorineindazole))phenyl)-*N*-(4-chloro-3–trifluoro-methyl phenyl) urea **97**.

Qu et al. [55] performed anticancer activities screening assays as well as cancer growth inhibitory studies on previously synthesized *N*-(4-(1-(4-chlorineindazole))phenyl)-*N*-(4-chloro-3-tri fluoromethyl phenyl) urea **97** (Figure 12). The results indicated that compound **97** could be a multi-kinases inhibitor targeting c-Kit, PDGFRβ and FLT3 with K_d_ values at 68.5 ± 9.5 nM, 140 ± 0 nM and 375 ± 15.3 nM, respectively. Compound **97** also possessed strong inhibition activities against 12 human cancer cells with IC_50_ values ranging from 1.12 to 6.84 μM. Especially, it exhibited strong inhibition on SK-MEL-3, NB-4, and SK-MEL-31 with IC_50_ values at 1.12 ± 0.06 μM, 1.34 ± 0.20 μM, and 1.77 ± 0.06 μM, respectively. Meanwhile, compound **97** effectively delayed the growth of cancer xenografts and safety to mice [56].

**Figure 13:**
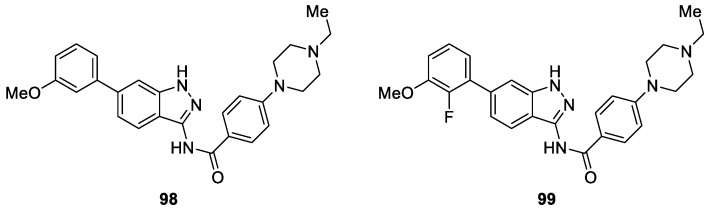
Chemical structures of 1*H*-indazol-3-amine derivatives **98** and **99**.

Li et al. [57] designed and synthesized a novel series of 1*H*-indazol-3-amine scaffold derivatives by utilizing scaffold hopping and molecular hybridization strategies aiming to develop potent FGFR inhibitors. The authors carried out an evaluation of their inhibitory activity against FGFR1. It was found that 6-(3-methoxyphenyl)-1*H*-indazol-3-amine derivative **98** was a promising FGFR1 inhibitor, exihibited good enzymatic inhibition (IC_50_ = 15.0 nM) and modest anti-proliferative activity (IC_50_ = 642.1 nM) (Figure 13). Further structural optimization revealed that compound **99** was identified as the most potent FGFR1 inhibitor with the best enzyme inhibitory (IC_50_ = 2.9 nM) and cellular activity (IC_50_ = 40.5 nM). The SAR studies revealed that *N*-ethylpiperazine group was important for enzyme inhibitory and cellular activity.

**Figure 14:**
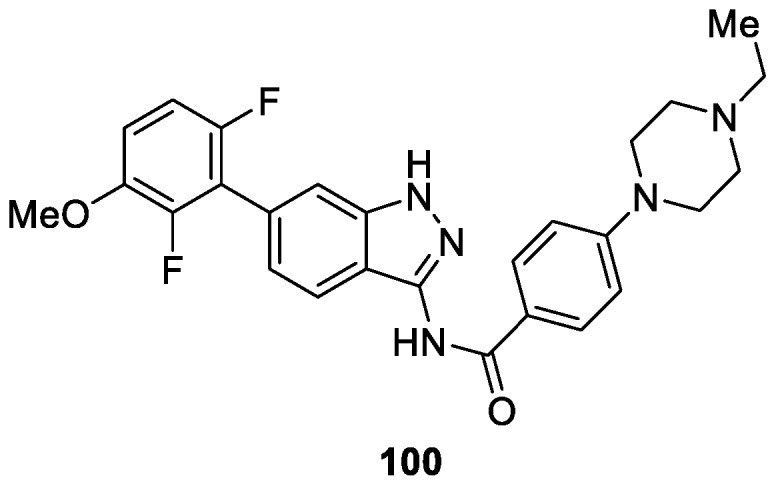
Chemical structure of 1*H*-indazol-3-amine derivative **100**.

As a continuation of their research to improve the cellular activity of hit compound **100** bearing an indazole scaffold, a series of new compounds harnessing fluorine substituents were designed, synthesized and evaluated as inhibitors of the above enzyme by Li et al. [58]. Among them, compound **100** containing 2,6-difluoro-3-methoxyphenyl group, possessed the most enzymatic and antiproliferative activities (FGFR1: IC_50_ value less than 4.1 nM, FGFR2: IC_50_ = 2.0 ± 0.8 nM, KG1 cell lines: IC_50_ = 25.3 ± 4.6 nM, SNU16 cell lines: IC_50_ = 77.4 ± 6.2 nM) (Figure 14).

**Figure 15:**
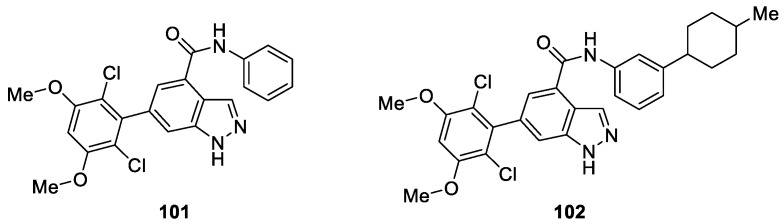
Chemical structures of 6-(2,6-dichloro-3,5-dimethoxyphenyl)-4-substituted-1*H*-indazole derivatives **101** and **102**.

Zhao et al. [59] disclosed novel 6-(2,6-dichloro-3,5-dimethoxyphenyl)-4-substituted-1*H*-indazole derivatives and assessed their potency as tyrosine kinase fibroblast growth factor receptor (FGFR) inhibitors for cancer therapy. The results indicated that compound **101** was a potent FGFR1 inhibitor, with good enzymatic inhibition (IC_50_ = 69.1 ± 19.8 nM) (Figure 15). According to the obtained structure activity relationship, the authors further designed and synthesized a new series of derivatives. 6-(2,6-Dichloro-3,5-dimethoxyphenyl)-*N*-(3-(4–methylpiperazin-1-yl)phenyl)-1*H*-indazole-4-carboxamide (**102**) exhibited the most potent FGFR1 inhibitory activity in the enzymatic assay (IC_50_ = 30.2 ± 1.9 nM).

**Figure 16:**
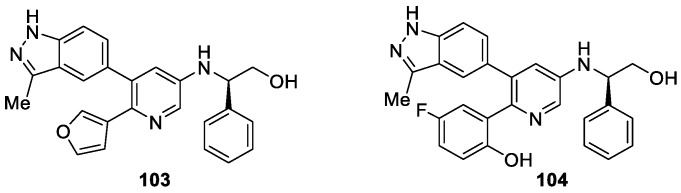
Chemical structures of pyridin-3-amine derivatives **103** and **104.**

Liu et al. [60] reported that the synthesis of pyridin-3-amine derivatives served as multi-targeted protein kinase inhibitors for the treatment of non-small cell lung cancer (NSCLC). First, they discovered compound **103** via in silico screening against fibroblast growth factor receptors (FGFR) and this was sequentially validated by in vitro experiments (IC_50_ value of 3.8 ± 0.5 μM against FGFR1). Aiming to improve the inhibition potency of compound **103** against FGFR1, the authors carried out a structure-based structural optimization, and then explored further to afford novel FGFR inhibitors (Figure 16). Among them, compound **104** displayed attractive inhibitory activity against FGFR1, 2 and 3 with IC_50_ values of 18.0, 1.6 and 27.5 nM, respectively. Notably, compound **104** also exhibited nanomolar level inhibition against several other NSCLC-related oncogenes kinases, such as RET, EGFR, EGFR/T790M/L858R, DDR2, and ALK. Significant antitumor activity (TGI = 66.1%) of compound **104** in FGFR-driven NCI-H1581 xenografts with good PK profiles was observed in vivo pharmacology evaluations.

**Figure 17:**
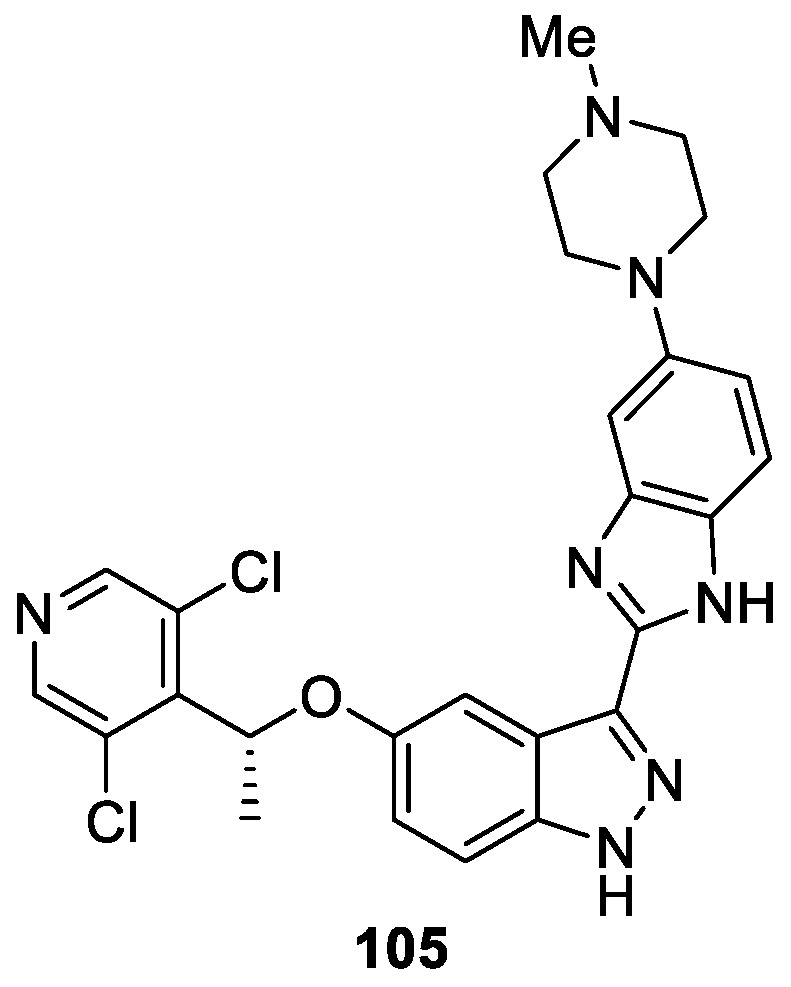
Chemical structure of 3-(5′-Substituted)–Benzimidazole-5-(1-(3,5-dichloropyridin-4-yl) ethoxy)-1*H*-indazole derivative **105**.

Duan et al. [61] reported the synthesis of novel 3-(5′-Substituted)–Benzimidazole-5-(1-(3,5-dichloropyridin-4-yl)ethoxy)-1*H*-indazole derivatives and evaluated for Fibroblast growth factor receptors (FGFRs) inhibitory activities. Among them, compound **105** was identified as a potent pan-FGFR inhibitor (FGFR1-4 IC_50_ values of 0.9, 2.0, 2.0, and 6.1 nM, respectively) (Figure 17). Moreover, compound **105** provided nearly complete inhibition of tumor growth (96.9% TGI) in NCI-H1581 (FGFR1-amplified) xenograft mice model at the dose of 10 mg/kg/qd via oral administration. 

**Figure 18:**
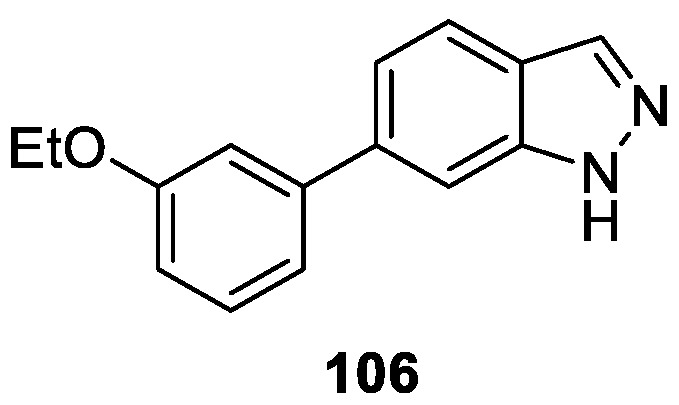
Chemical structure of 1*H*-indazole derivative **106**.

Fishwick et al. [62] discovered a novel series of 1*H*-indazole-based derivatives for the inhibition of Fibroblast growth factor receptors (FGFRs) kinases using fragment-led de novo design. Biological evaluation indicated that these 1*H*-indazole-based derivatives inhibited FGFR1-3 in the range of 0.8–90 μM with excellent ligand (LE) efficiencies of 0.30–0.48. Among them, compound **106** was identified as the most active inhibitor (FGFR1-3 IC_50_ values of 2.0 ± 0.4, 0.8 ± 0.3 and 4.5 ± 1.6 μM, respectively) (Figure 18).

**Figure 19:**
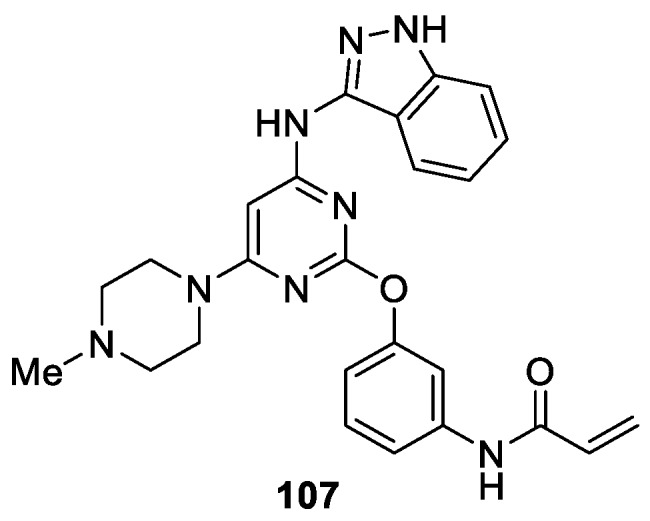
Chemical structure of 1*H*-indazole derivative **107**.

Rauh et al. [63] described a structure-based approach, rationalized by subsequent computational analysis of conformational ligand ensembles in solution, to design 1*H*-indazole analogues as irreversible and mutant-selective EGFR inhibitors based on systematic optimization of 80 NSCLC cell lines against approximately 1500 compounds. The vitro assay study revealed that among the synthesized and tested compounds, **107** was the most active compound against the L858R/T790M double mutant of EGFR with IC_50_ values of 0.07 μM. Meanwhile, **107** exhibited moderate effects on the activating and drug resistant cell lines HCC827 and H1975 (GI_50_ = 2.5 μM and 9.8 μM, respectively) (Figure 19).

**Figure 20:**
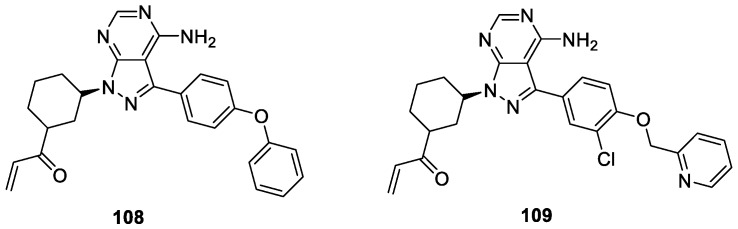
Chemical structures of 1*H*-indazole derivatives **108** and **109**.

A series of 1*H*-indazole derivatives were synthesized using structure-guided drug design from lead compound **108** by Liu et al. [64] and evaluated for their epidermal growth factor receptor (EGFR) kinase activity. The results indicated that compound **109** displayed strong potencies against EGFR T790M and EGFR kinases with IC_50_ values of 5.3 and 8.3 nM, respectively (Figure 20). In particular, compound **109** displayed strong antiproliferative effects against EGFR mutant-driven non-small cell lung cancer (NSCLC) cell lines such as H1975, PC9, HCC827, and H3255 without obvious toxicity.

**Figure 21:**
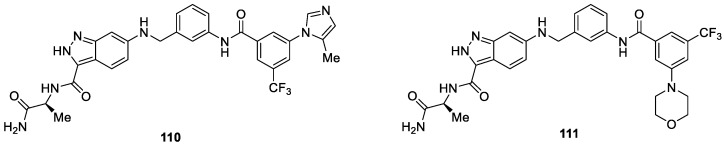
Chemical structures of 3-carboxamido-2*H*-indazole-6-arylamide derivatives **110** and **111**.

Hah et al. [65] reported the synthesis of novel 3-carboxamido-2*H*-indazole-6-arylamide derivatives as selective CRAF inhibitors. The compounds were evaluated for antiproliferative activity against the WM3629 melanoma cell line. Results revealed that most of the compounds displayed potent antiproliferative activity against the WM3629 melanoma cell line. In particular, compounds **110** and **111** exhibited superior selectivity (Figure 21). Compound **110** was found to be the most promising inhibitor against the WM3629 melanoma cell line with IC_50_ value of 38.6 nM, which also showed enzymatic activities against wild-type BRAF and BRAF (V599E) kinase with IC_50_ values of 9.45 and 7.82 μM, respectively.

**Figure 22:**
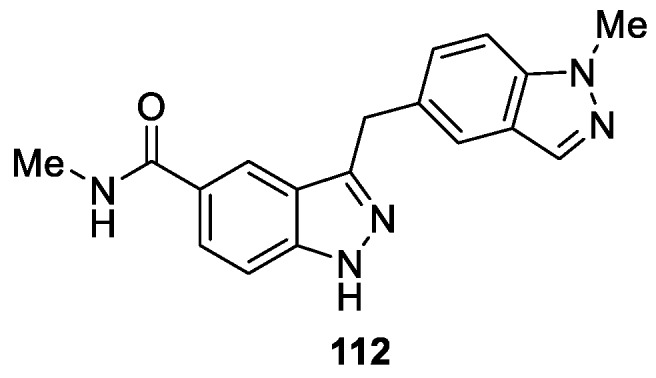
Chemical structure of 3-benzylindazoles derivative **112**.

Schiemann et al. [66] synthesized a series of 3-benzylindazoles analogues based on a high-throughput screening (HTS) campaign sampling the Merck compound collection, which were tested as potent and selective CDK8 inhibitors with high affinity to CDK19. Medicinal chemistry optimization allowed them to identify inhibitors with improved potency, physicochemical properties and oral pharmacokinetics. Compound **112** displayed promising selectivity against CDK8 with IC_50_ value of 53 nM (Figure 22).

**Figure 23:**
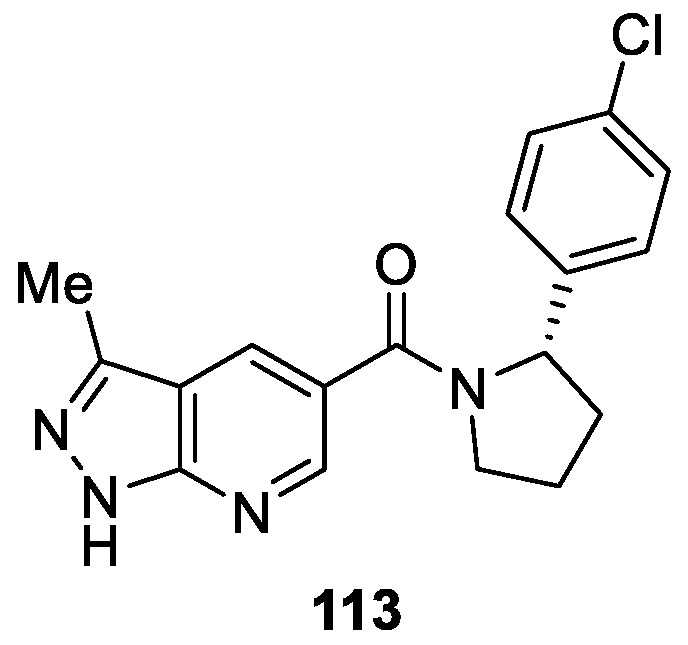
Chemical structure of imidazo-thiadiazole derivative **113**.

Czodrowski et al. [67] described a series of imidazo-thiadiazole derivatives as CDK8 inhibitors based on a Forster resonance energy transfer (FRET)-based Lanthascreen binding competition assay. In several optimization cycles, compounds **113** displayed excellent kinase selectivity (CDK8 IC_50_ = 2.6 nM), biochemical and cellular potency, microsomal stability (Figure 23). In particular, compound **113** demonstrated reduction of tumor growth rates of established human SW620 colorectal carcinoma xenografts using two different oral dosing schedules.

**Figure 24:**
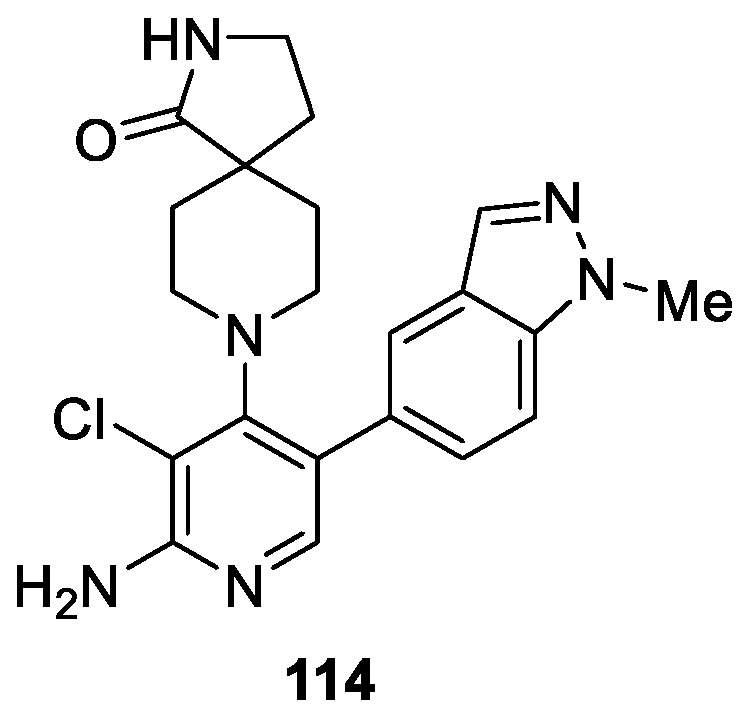
Chemical structure of 1*H*-indazole derivative **114**.

Mallinger et al. [68] disclosed a novel series of 1*H*-indazole derivatives and the application of physicochemical property analyses to successfully reduce in vivo metabolic clearance, minimize transporter-mediated biliary elimination while maintaining acceptable aqueous solubility. The results indicated that compound **114** was a potent selective, and orally bioavailable inhibitor of CDK8 (IC_50_ = 2.3 ± 0.8 nM) with equipotent affinity for CDK19 (IC_50_ = 2.6 ± 0.4 nM) (Figure 24). According to the assay results, compound **114** afforded the optimal compromise of in vitro biochemical, pharmacokinetic, and physicochemical properties and was suitable for progression to animal models of cancer.

**Figure 25:**
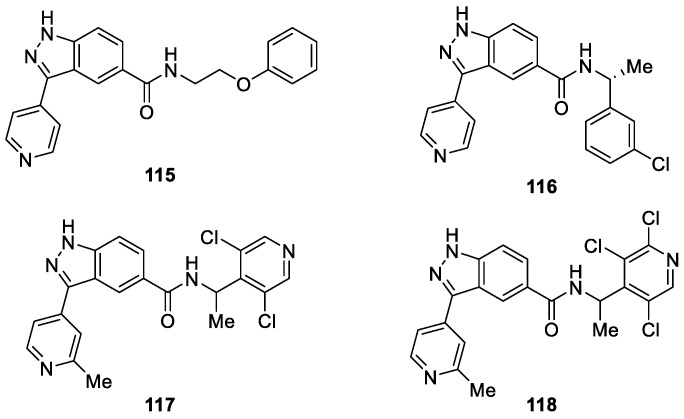
Chemical structures of 1*H*-indazole amide derivatives **115**–**118**.

A series of 1*H*-indazole amide derivatives were synthesized using structure-guided and knowledge-based design from lead compound **115** by Cao et al. [69] and evaluated for their extracellular signal-regulated kinase1/2 (ERK1/2) activity. The results indicated that most advanced compounds **116**, **117** and **118** showed both very good enzymatic and cellular activity toward ERK1/2 and HT29 cell lines with IC_50_ values ranging between 9.3 ± 3.2~25.8 ± 2.3 nM, 0.9 ± 0.1~6.1 ± 1.1 μM, respectively (Figure 25).

**Figure 26:**
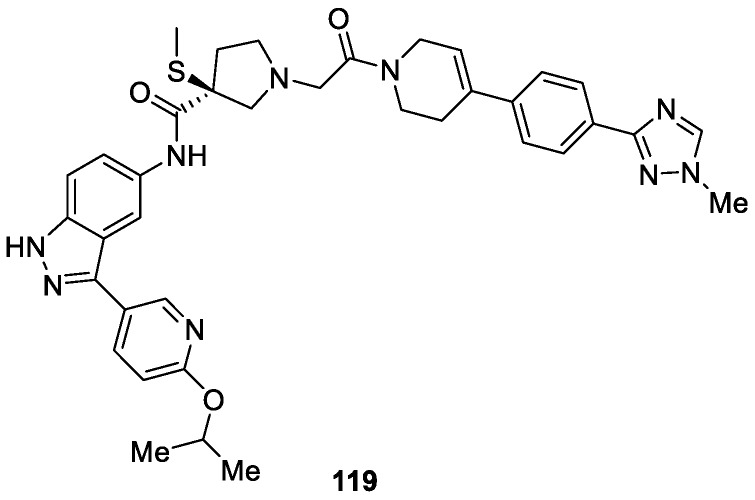
Chemical structure of 3(S)-thiomethyl pyrrolidine-1*H*-indazole derivative **119**.

Babu Boga et al. [70] developed a series of novel 3(S)-thiomethyl pyrrolidine-1*H*-indazole derivatives aiming to identify new and safe compounds as ERK inhibitors for treatment of cancer. Among all the tested compounds, compound **119** displayed excellent potency, high ERK 1/2 selectivity and dual mechanisms of action inhibition (IC_50_ = 20 and 7 nM, respectively) (Figure 26). Additionally, the detailed pharmacological and clinical evaluation demonstrated that compound **119** was well tolerated up to 400 mg twice daily and exhibited antitumor activity in patients with BRAFV600-mutant melanoma.

**Figure 27:**
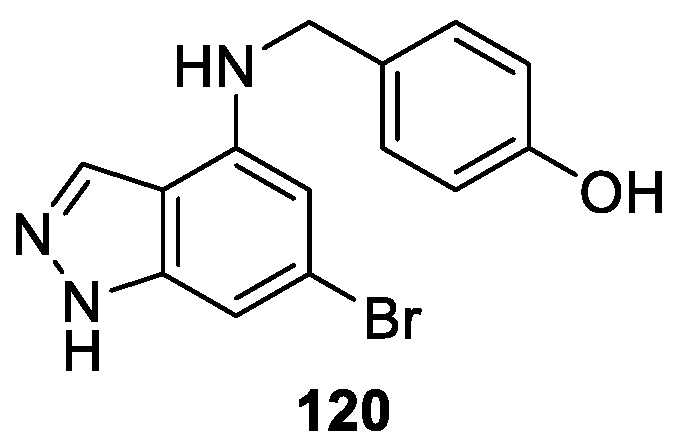
Chemical structure of 4-(((6-Bromo-1*H*-indazol-4-yl)amino)methyl)phenol **120**.

Qian et al. [71] synthesized a novel series of 1*H*-indazole derivatives with disubstituent groups at both 4-position and 6-position. The authors carried out IDO1 inhibition assay using three inhibitory concentrations. The results revealed that some compounds displayed remarkable IDO1 inhibitory activities. Among all of the tested coumponds, compound **120** exhibited the most IDO1 inhibitory activity with an IC_50_ value of 5.3 μM (Figure 27). The docking model indicated that the effective interactions of 1*H*-indazoles motif with ferrous ion of heme and hydrophobic pocket A and B ensured the IDO1 inhibitory activities, which demonstrated that 1*H*-indazole structure was a novel key pharmacophore with potent IDO1 inhibitory activity. The structure-activity relationships (SARs) analysis of the synthesized derivatives suggested that the substituent groups at both 4-position and 6-position of 1*H*-indazole scaffold played a crucial role in the IDO1 inhibition.

**Figure 28:**
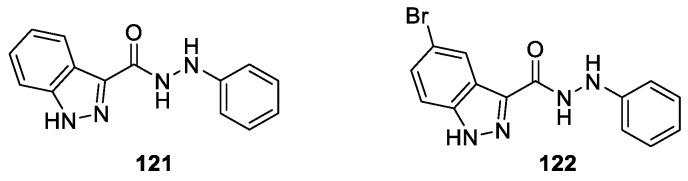
Chemical structures of 3-substituted 1*H*-indazoles **121** and **122**.

Manna et al. [72] described the synthesis of a series of 3-substituted 1*H*-indazoles which were investigated for their IDO1 enzyme inhibition efficiencies. The studies revealed that, among the synthesized and tested derivatives, **121** and **122** possessed the most potent inhibitory activity with IC_50_ values of 720 and 770 nM, respectively (Figure 28). According to SAR studies, the presence of 1*H*-indazole ring and suitably substituted carbohydrazide moiety at the C3 position of the indazole ring played a crucial role for their strong inhibitory activities in vitro. 

**Figure 29:**
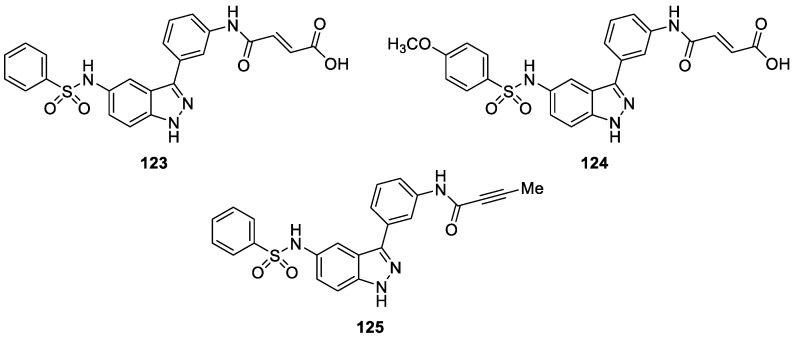
Chemical structures of 1*H*-indazole derivatives **123**–**125**.

Hsieh et al. [73] described the synthesis of indazole-based derivatives by utilizing *in silico* fragment-based approach and knowledge-based drug design and evaluated them for Aurora kinase activity. The study revealed that, among the optimized derivatives, compounds **123** (dual Aurora A and B), **124** (Aurora B selective) and **125** (Aurora A selective) provided sub-type kinase selectivity (Figure 29). Furthermore, compounds **123** appeared to be the most potent dual Aurora A and B inhibitor (IC_50_ = 0.026, 0.015 μM, respectively). Docking analysis revealed that compound **123** formed hydrogen bonds with particular targeting residues Glu211, Ala213, Lys141, Thr217 and Arg220 in Aurora kinase binding pocket.

**Figure 30:**
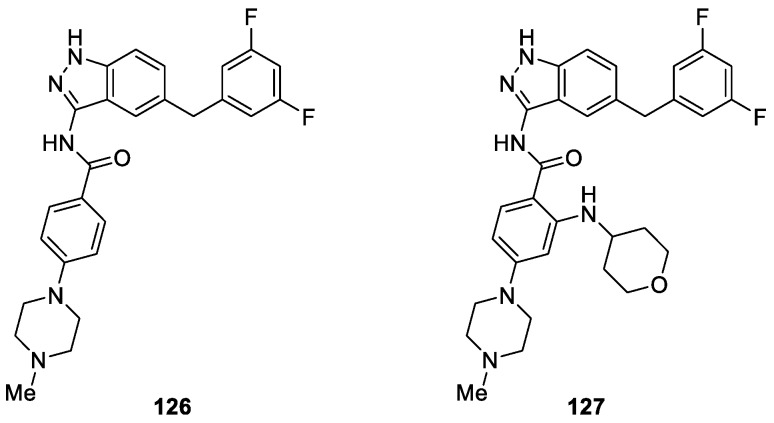
Chemical structures of 3-aminoindazole derivatives **126** and **127** as ALK inhibitors.

Menichincheri et al. [74] used 3-amino-5-substituted indazole **126** as a promising starting point for further investigation and obtained novel 3-aminoindazole derivatives. The synthesized compounds were tested for their enzyme inhibition and antiproliferative activities. Results revealed that compound **127** (entrectinib) showed highest activity against anaplastic lymphoma kinase (ALK) with an IC_50_ value of 12 nM (Figure 30). Moreover, compound **127** caused potent inhibition of the closely related tyrosine kinases ROS1 and TRKs with nanomolar activity and it was recently used in phase I/II clinical trial for the treatment of patients affected by ALK-, ROS1- and TRK-dependent tumors [75,76].

**Figure 31:**
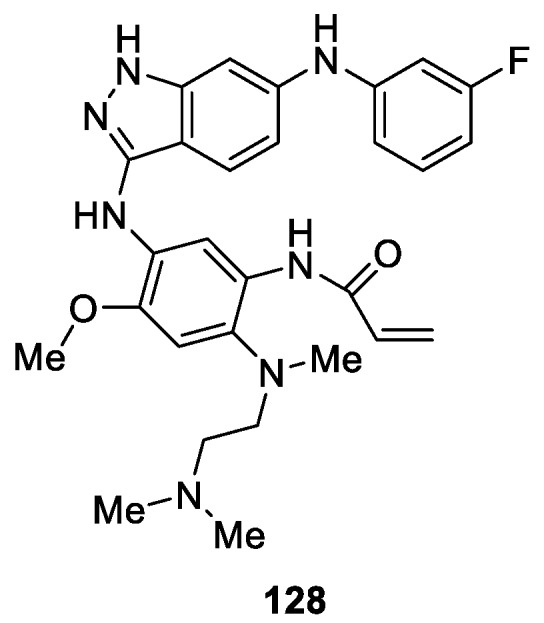
Chemical structure of indazole-based EGFR inhibitor **128**.

Rauh et al. [77] reported the synthesis of novel selective indazole-based derivatives as potential inhibitors of mutated drug resistant EGFR-L858R/T790M and covalently alkylate Cys797. The assay resulted in a promising compound **128** (Figure 31), which displayed inhibitory activity against H1975 and HCC827 cell lines with the EC_50_ values of 191 nM and 22 nM, respectively, while sparing the wild type with a EC_50_ value of 3103 nM in A431 cells.

**Figure 32:**
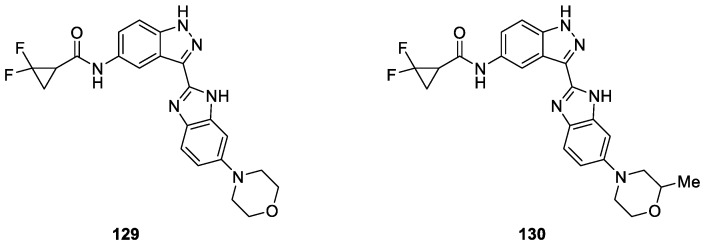
Chemical structures of 1*H*-benzo[*d*]imidazol-2-yl)-1*H*-indazol derivatives **129** and **130**.

Vankayalapati et al. [78] prepared a novel class of 1*H*-benzo[d]imidazol-2-yl)-1*H*-indazol derivatives as phosphoinositide-dependent kinase-1 (PDK1) inhibitors based on a fragment-based, structure-assisted approach. The preliminary biological results led to the identification of the two most active compounds, namely compounds **129** and **130** (Figure 32). These lead compounds exhibited potent PDK1 activity with IC_50_ values of 80 and 90 nM, respectively.

**Figure 33:**
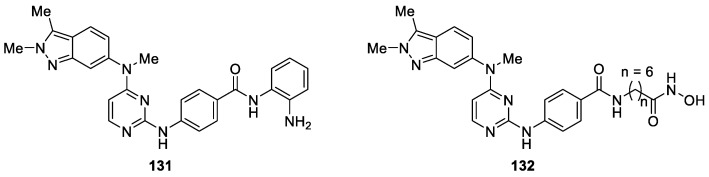
Chemical structures of 2*H*-indazole derivatives **131** and **132**.

Recently, Zhang et al. [79] developed a novel series of pazopanib hybrids as polypharmacological antitumor agents based on the crosstalk between histone deacetylases (HDACs) and vascular endothelial growth factor (VEGF) pathway. Among these compounds, *ortho*-aminoanilide based compound **131** and pazopanib-based hydroxamic acid **132** were identified as the most potent histone deacetylases (HDACs) inhibitors with IC_50_ values of 4.6 and 0.0033 μM, respectively (Figure 33). It is worth noting that compound **131** exhibited more potent antiproliferative activity against HT-29 than both SAHA and MS-275, while compound **132** was slightly more potent than MS-275. Furthermore, compounds **131** and **132** displayed comparable vascular endothelial growth factor receptor (VEGFR) signaling pathway potencies to pazopanib with IC_50_ values of 37 and 46 nM, respectively. Additionally, compound **131** possessed desirable pharmacokinetic profiles with oral bioavailability of 72% in SD rats and considerable in vivo antitumor efficacy in a human colorectal adenocarcinoma (HT-29) xenograft model [80].

**Figure 34:**
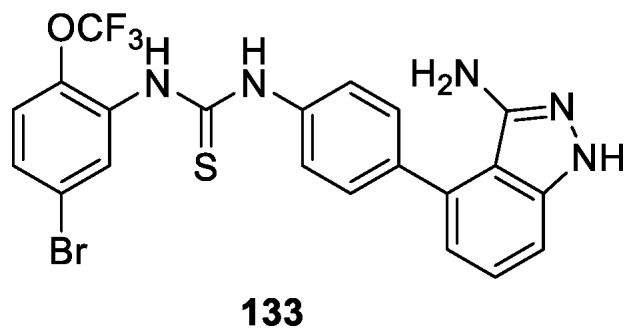
Chemical structure of 1*H*-indazole-3-amine derivative **133**.

Zhang et al. [81] designed and prepared three classes of multi-target inhibitors based on the extensive sequence homology along the kinase domain of angiogenic RTKs. Biological evaluation indicated that these multi-target inhibitors exhibited considerable potential as novel anti-angiogeneic and anticancer agents. Among them, compound **133** showed the most potent multi-target RTKs inhibitors with IC_50_ values of 3.45 nM (VEGFR-2), 2.13 nM (Tie-2), and 4.71 nM (EphB4), respectively (Figure 34). Additionally, compound **133** also showed inhibition on the viability of human umbilical vein endothelial cells and antiproliferation against a broad spectrum of cancer cells.

**Figure 35:**
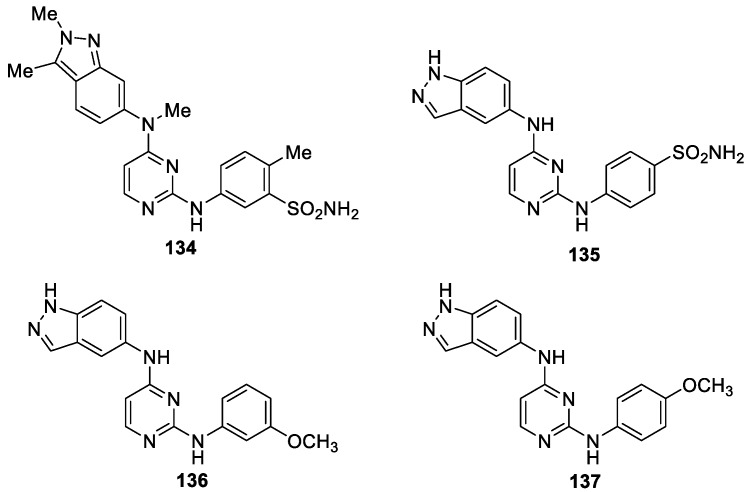
Chemical structures of indazole–pyrimidine derivatives **134**–**137**.

Abouzid et al. [82] reported the synthesis of three series of novel indazole-pyrimidine based derivatives based on the development of derivatives of pazopanib **134** (VEGFR-2 IC_50_ = 30 nM) (Figure 35). The synthesized compounds were tested for their VEGFR-2 kinase inhibitory activity and selected compounds were evaluated for their inhibitory activity against the NCI-60 cancer cell line. Among the tested derivatives, compound **135** showed the highest potency inhibitory activity against VEGFR-2 with the IC_50_ value of 24.5 nM and cellular anti-angiogenic activity against the human umbilical vein endothelial cells (HUVEC) cell line with the IC_50_ value of 1.37 μM. In addition, compound **136** exerted nanomolar GI_50_ values against several cell lines: CCRF-CEM (901 nM), MOLT-4 (525 nM) and CAKI-1 (992 nM). Compound **137** showed one-digit micromolar activity against the whole panel of cell lines ranging from 1.55 μM to 7.4 μM.

**Figure 36:**
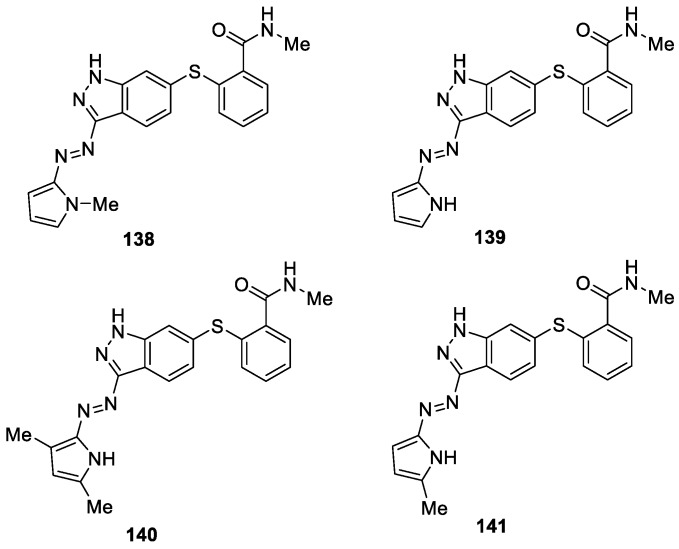
Chemical structures of 1*H*-indazole derivatives **138**–**141**.

Dong et al. [83] prepared a novel series of 1*H*-indazole derivatives and investigated against VEGFR-2 kinase inhibitory activities and anti-proliferative activities in vitro. Biological activity results indicated that most of the nascent compounds under investigation exhibited significant VEGFR-2 kinase inhibitory activity. Among them, **138**, **139**, **140**, and **141** exhibited more potent inhibitory activities and favorable anti-proliferative activities at the concentration of 10 μM with IC_50_ values of 5.03 μM (SD = 1.13, n = 3), 5.73 μM (SD = 1.30, n = 3), 2.18 μM (SD = 1.43, n = 3), and 2.15 μM (SD = 1.46, n = 3), respectively (Figure 36).

**Figure 37:**
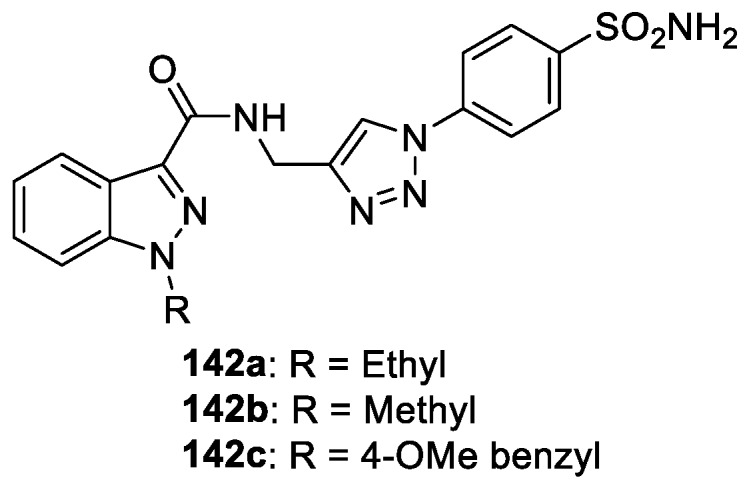
Chemical structures of indazole-3-carboxamide derivatives **142a**–**c**.

Arifuddin et al. [84] reported a novel series of sulfocoumarin/coumarin/4-sulfamoylphenyl bearing indazole-3-carboxamide hybrids. The synthesized compounds were evaluated in vitro as inhibitors against four relevant human carbonic anhydrases (hCAs), comprising the cytosolic and ubiquitous isozymes hCA I and II as well as the transmembrane hCA IX and hCA XII. Among them, compounds **142a**, **142b** and **142c** potentially inhibited tumor associated, hypoxia induced isoforms hCA IX with k_I_s 1.8, 2.3, and 2.0 nM, respectively (Figure 37).

**Figure 38:**
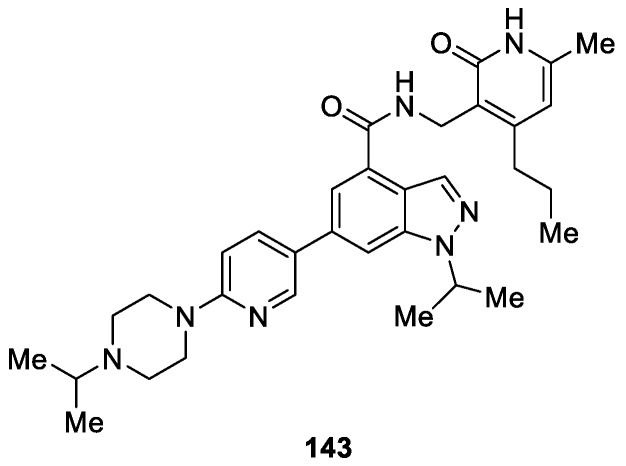
Chemical structure of 1*H*-indazole derivative **143**.

Jin et al. [85] report SAR studies that focus on modifications to various regions of the enhancer of zeste homologue 2/1 (EZH2/1) inhibitor UNC1999 (**143**) to investigate the impact of the structural changes on EZH2 and EZH1 inhibition and selectivity (Figure 38). The SAR studies demonstrated that the two methyl groups at the 4 and 6 positions of the pyridone ring showed the importance of inhibiting these two enzymes, especially EZH2. In addition, the indazole ring was the best among the heterocyclic rings and various substituents at the N-1 position of this ring system had stronger effects on EZH1 potency than EZH2 potency.

**Figure 39:**
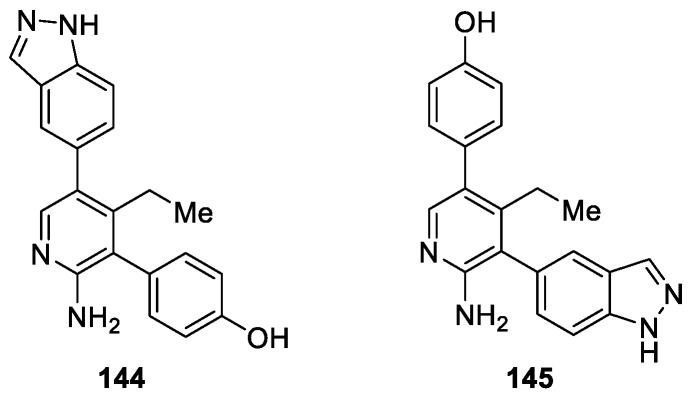
Chemical structures of 1*H*-indazole derivatives **144** and **145**.

Di Lello et al. [86] adopted fragment-based lead discovery (FBLD) by NMR combined with virtual screening and re-mining of biochemical high-throughput screening (HTS) hits led to the discovery of a series of ligands that bind in the “palm” region of the catalytic domain of ubiquitin specific protease 7 (USP7) and inhibit its catalytic activity. These ligands were then optimized by structure-based design to yield cell-active molecules with reasonable physical properties. Among them, compound **144** and **145** displayed the most promising activity against USP7 with IC_50_ values of 0.75 and 0.61μM, respectively (Figure 39). MDM2 MSD cell-based assay results showed that compound **145** was the most potent analog with an EC_50_ value of 0.30 μM. Furthermore, compound **145** decreased cell viability via both apoptosis and cell cycle arrest.

**Figure 40:**
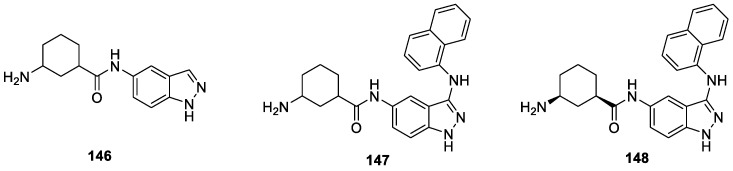
Chemical structures of 1*H*-indazole derivatives **146**–**148**.

Roush et al. [87] using an in silico HTS campaign utilizing a published X-ray structure of ULK1 and the electronic coordinates of an in-house chemical library, identified compound **146** as a moderately active Unc-51-like kinase 1 (ULK1) inhibitor (IC_50_ = 22.4 μM). Further optimization of compound **146** using structure-guided rational drug design then resulted in significantly more potent ULK1 inhibitors. Among them, compounds **147** and **148** exhibited strong inhibition on ULK1 with IC_50_ values of 11 and 45 nM, respectively (Figure 40). SAR efforts confirmed that 3-aminocyclohexane substituent played a crucial role in ULK1 inhibition.

### 3.2. Antimicrobial Activity

**Figure 41:**
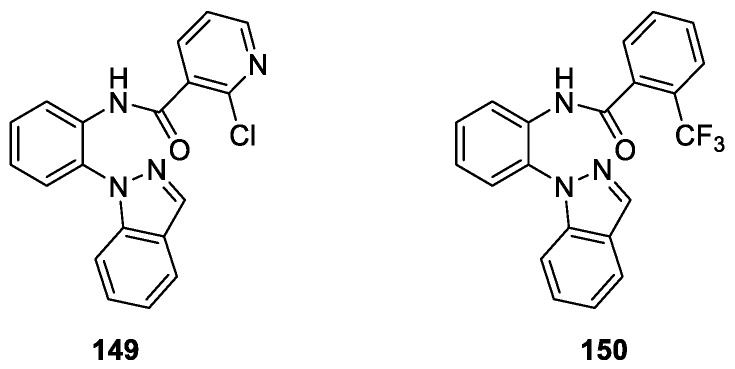
Chemical structures of 1*H*-indazole derivatives **149** and **150**.

Qin et al. [88] described the synthesis of a novel series of aromatic carboxylic acid amides containing 1*H*-indazole moiety based on a bioisosterism approach and evaluation of their activities against six phytopathogenic fungi by an in vitro mycelia growth inhibition assay. The preliminary biological results demonstrated that all of the target molecules displayed moderate to good activity against the six kinds of fungi. Among them, compounds **149** and **150** exhibited higher inhibition activity than others (Figure 41). In particular, *N*-(2-(1*H*-indazol-1-yl)phenyl)-2-(trifluoromethyl)-benzamide (**150**) exhibited the highest antifungal activity against *Pythium aphanidermatum* (EC_50_ = 16.75 μg/mL) and *Rhizoctonia solani* (EC_50_ = 19.19 μg/mL), respectively. The molecular docking studies indicated that the fluorine and the carbonyl oxygen atom of **150** formed hydrogen bonds with the hydroxyl hydrogens of TYR58 and TRP173.

**Figure 42:**
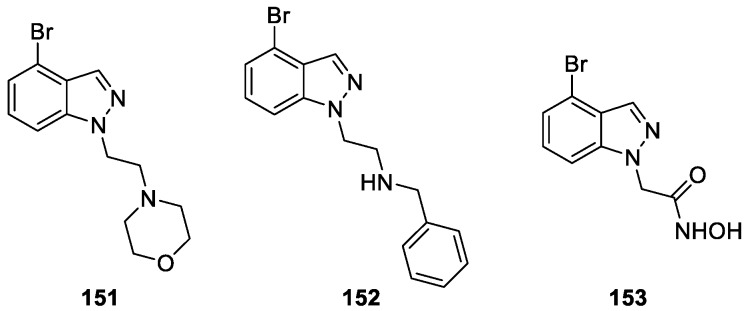
Chemical structures of 4-bromo-1*H*-indazole derivatives **151**, **152** and **153**.

Ma et al. [89] developed a series of novel 4-bromo-1*H*-indazole derivatives aiming to identify new and safe compounds as filamentous temperature-sensitive protein Z (FtsZ) inhibitors. The authors performed an evaluation of their antibacterial activity and cell inhibitory activity against various phenotypes of Gram-positive and Gram-negative bacteria. Among all the tested compounds, compounds **152** and **153** exhibited more potent activity than 3-methoxybenzamide (3-MBA) against penicillin-resistant staphylococcus aureus (Figure 42). Particularly, compound **151** presented the best activity with an MIC value of 4mg/mL against S. pyogenes PS in the tested compounds.

**Figure 43:**
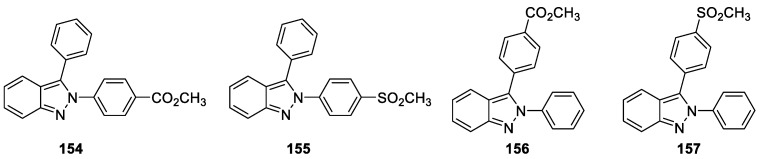
Chemical structures of 2,3-diphenyl-2*H*-indazole derivatives **154**–**157**.

A new set of 2*H*-indazole derivatives were studied for their activities against selected intestinal and vaginal pathogens, including the protozoa Giardia intestinalis, Entamoeba histolytica, and Trichomonas vaginalis; the bacteria Escherichia coli and Salmonella enterica serovar Typhi; and the yeasts Candida albicans and Candida glabrata by Pérez-Villanueva et al. [90]. Biological evaluations revealed that most of the synthesized compounds showed more potent antiprotozoal activity than metronidazole. Furthermore, compounds **154** and **155** inhibited in vitro growth of C. albicans and C. glabrata with the same minimum inhibitory concentration (MIC) (Figure 43). In addition, compounds **154**, **155**, **156**, and **157** were identified as anti-inflammatory agents and displayed in vitro inhibitory activity against COX-2 (36–50%, at 10 μM).

### 3.3. Anti-Diabetic Agents

**Figure 44:**
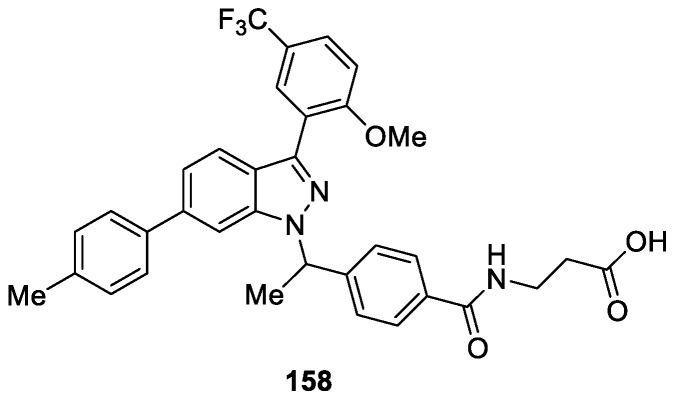
Chemical structure of 1*H*-indazole derivative **158**.

A novel series of indazole-based compounds were designed and synthesized by Lin et al. [91] as glucagon receptor antagonists (GRAs) for treatment of type 2 diabetes mellitus. Among them, compound **158** was identified to be orally active in blunting glucagon induced glucose excursion in an acute glucagon challenge model in glucagon receptor humanized (hGCGR) mice at 1, 3 and 10 mg/kg (mpk), and significantly lowered acute glucose levels in hGCGR ob/ob mice at 3 mpk dose (Figure 44). Structure-activity relationship (SAR) studies revealed that aryl groups on the C3 and C6 positions of the indazole core were crucial for inhibitory activities.

**Figure 45:**
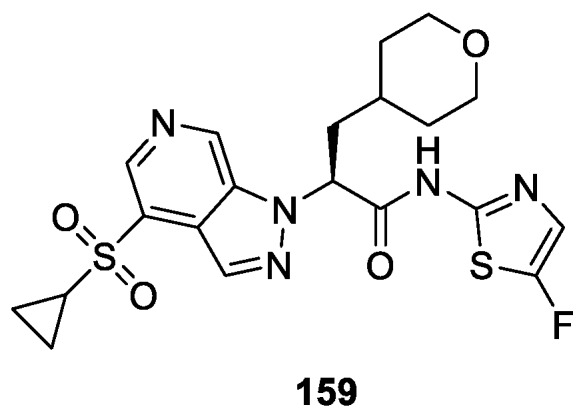
Chemical structure of 1,4-disubstituted-1*H*-indazole derivative **159**.

Cheruvallath et al. [92] discovered a novel class of 1,4-disubstituted indazole derivatives as the potent Glucokinase activators using scaffold morphing and structure guided medicinal chemistry approach. The anti-diabetic oral glucose tolerance test (OGTT) demonstrated that compound **159** exhibited promising hERG (human Ether-a-go-go Related Gene) inhibitory activity with EC_50_ values of 0.08 μM (Figure 45). It was further established that compound **159** combined the best balance of GK activation and in vitro DMPK properties.

**Figure 46:**
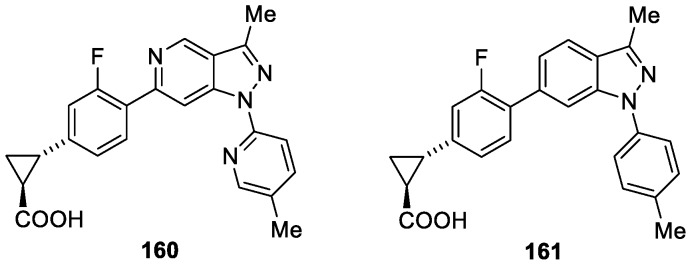
Chemical structures of 1*H*-indazole derivatives **160** and **161**.

McCoull et al. [93] identified an indazole-6-phenylcyclopropylcarboxylic acid series of GPR120 agonists and (S,S)-cyclopropylcarboxylic acid series of GPR40 agonists. Among them, compounds **160** and **161** exhibited potent GPR120 inhibition activity with EC_50_ values of 0.74 and 0.36 μM, respectively (Figure 46). Furthermore, compounds **160** and **161** were progressed to in vivo studies and demonstrated significant reduction in blood glucose excursion in response to a glucose challenge. Taking all these data together, the two compounds were excellent in vivo for exploring the agonist pharmacology of the GPR120.

### 3.4. Anti-Inflammatory Activity

**Figure 47:**
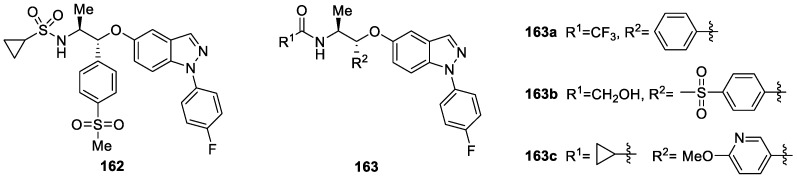
Chemical structures of 1*H*-indazole ether derivatives **162** and **163a**–**c**.

Hemmerling et al. [94] adopted a structure-based design approach to obtain a novel class of indazole ether based molecular scaffolds and evaluated their glucocorticoid receptor (GR) modulate activities. The results indicated that several examples displayed efficacy in a cellular transrepression assay at picomolar concentrations. Additionally, compound **162**, **163a**, **163b** and **163c** were investigated in the SCW rat PD model where in vitro potency translated well into in vivo efficacy (Figure 47), which showed that they were potential GR modulators for treatment of inflammatory diseases. 

**Figure 48:**
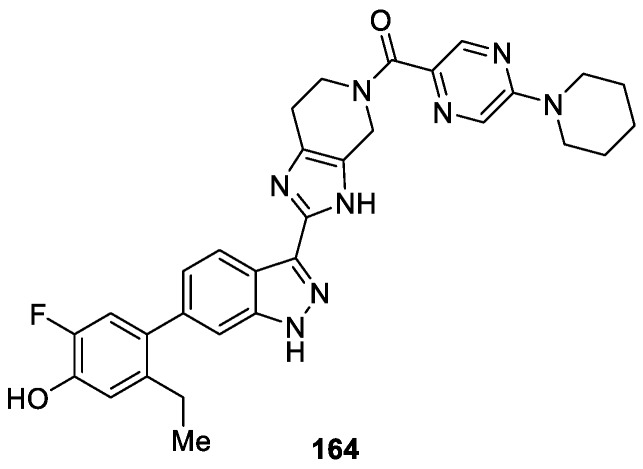
Chemical structure of 1*H*-indazole analogue **164**.

Jones et al. [95] discovered a series of novel 1*H*-indazoles analogues as selective pan-Janus kinase (JAK) inhibitors with a type 1.5 binding mode by means of structure-based computational method. After optimizing the synthesized compounds for potency and increased duration of action commensurate with inhaled or topical delivery, the authors identified a potent inhibitor **164** (PF-06263276) (Figure 48), which displayed good cellular JAK inhibitory activities and was potentially well suited for use as an inhaled or topical therapy to treat inflammatory diseases.

**Figure 49:**
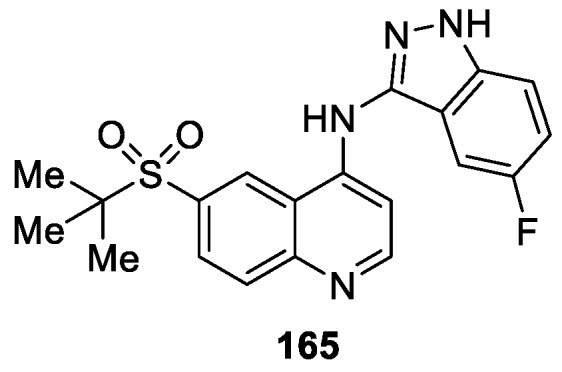
Chemical structure of 6-(*tert*-Butylsulfonyl)-*N*-(5-fluoro-1*H*-indazol-3-yl)quinolin-4-amine **165**.

Casillas et al. [96] identified and characterized 6-(*tert*-Butylsulfonyl)-*N*-(5-fluoro-1*H*-indazol-3-yl)quinolin-4-amine (**165**) as a potent and highly selective inhibitor of RIP2 kinase (IC_50_ = 5 nM) for the treatment of chronic inflammatory diseases (Figure 49). Compound **165** potently and dose dependently inhibited MDP stimulated tumor necrosis factor-alpha (TNFα) production with an IC_50_ = 8 nM. Compound **165** also inhibited the increase in serum levels of KC following administration of the NOD1 ligand, FK156 in mice with a single oral dose of 10 mg/kg. Moreover, in human biopsy assay, **165** significantly inhibited both TNFα and IL-6 production in a concentration-dependent fashion in both Crohn’s disease (CD) and ulcerative colitis (UC) samples.

**Figure 50:**
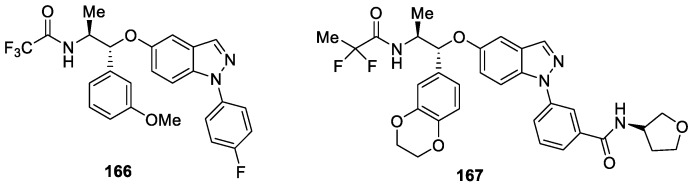
Chemical structures of 6 indazole ether-based derivatives **166** and **167**.

Hemmerling et al. [97] developed a class of potent, nonsteroidal, selective indazole ether-based glucocorticoid receptor modulators (SGRMs) for the treatment of airway inflammation. The authors developed a soft-drug strategy to deliver the first inhaled candidate **166**, which potently inhibited a Sephadex-induced lung edema in a dose-dependent manner with an ED_50_ value of 1.2 μg/kg (Figure 50). Further optimization of the inhaled drug properties provided a second, equally potent, candidate **167**, which achieved the requisite low solubility profile but retained the potency of compound **166** and demonstrated a robust local anti-inflammatory effect in rat lung airways at the same dose.

**Figure 51:**
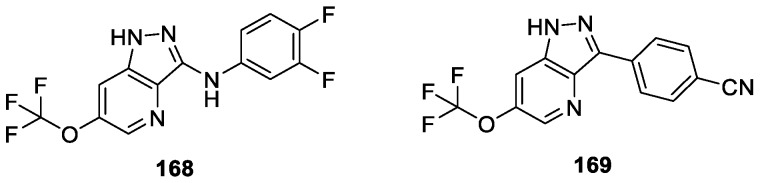
Chemical structures of 1*H*-indazole derivatives **168** and **169**.

Pryde et al. [98] reported a series of novel 1*H*-indazole derivatives as transient receptor potential ankyrin-repeat 1 (TRPA1) antagonists. The authors investigated the derivatives’s physical properties and in vitro drug metabolism and pharmacokinetics (DMPK) profiles. Compounds **168** and **169** showed significant activity against hTRPA1 with IC_50_ values of 20 and 686 nM, respectively, when administered either systemically or topically (Figure 51). Furthermore, the computational docking model proposed that compound **169** bounded in the S5 region of TRPA1 channel.

**Figure 52:**
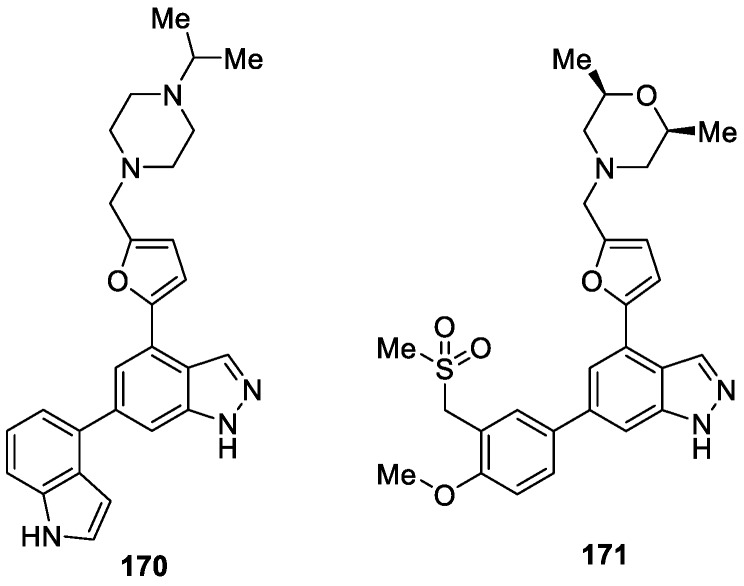
Chemical structures of 4,6-disubstituted-1*H*-indazole derivatives **170** and **171**.

Hamblin et al. [99] synthesized a novel series of 4,6-disubstituted-1*H*-indazole derivatives as selective phosphoinositide 3-kinase delta (PI3Kδ) inhibitors for the treatment of respiratory disease. The authors discovered compounds **170** and **171** via a structure-based design approach (Figure 52). Compounds **170** and **171** were both highly selective against PI3Kδ with p*K*_i_ values of 9.9 and 10.1, respectively. Moreover, in the brown Norway rat acute OVA model of Th2 driven inflammation in the lungs of rats, compounds **170** and **171** were shown to protect against eosinophil recruitment with ED_50_ of 67 and 35 μg/kg, respectively.

**Figure 53:**
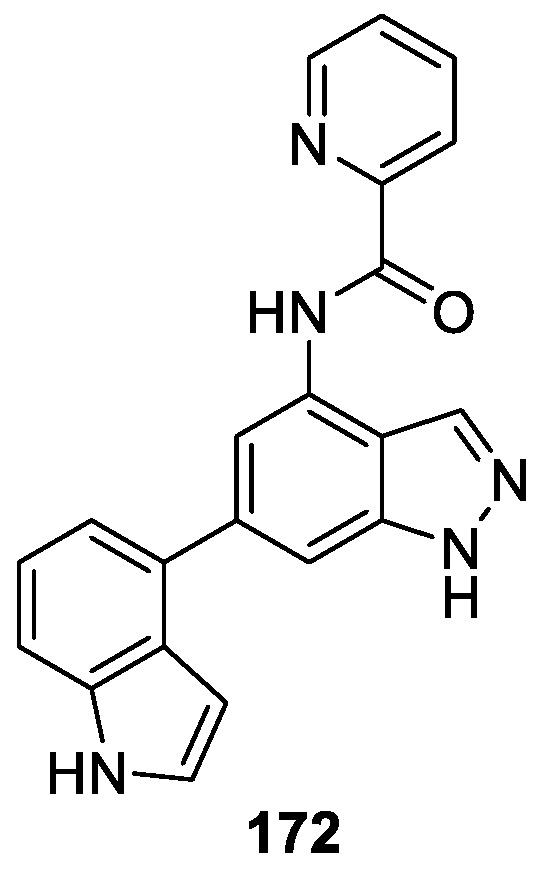
Chemical structure of 4,6-disubstituted-1*H*-indazole derivative **172**.

Henley et al. [100] described a novel, potent, and selective series of 4,6-disubstituted-1*H*-indazole derivatives and subsequent exploration of the structure-activity relationship led to the discovery of phosphoinositide 3-kinase delta (PI3Kδ) inhibitor for the treatment of inflammatory diseases. Among them, compound **172** showed significant PI3Kδ activity (pIC_50_ = 7.0), specifically against the other PI3K isoforms (α pIC_50_ = 5.0, ß pIC_50_ = 5.2, γ pIC_50_ = 5.2) (Figure 53).

**Figure 54:**
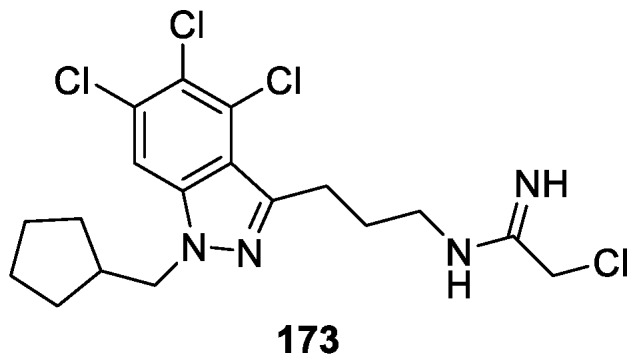
Chemical structure of *N*-alkylated-1*H*-indazole derivative **173**.

Ellman et al. [101] prepared and evaluated a novel series of *N*-alkylated indazole chloroacetamidine derivatives as potential protein arginine deiminase 4 (PAD4) inhibitors. Derivatization around the indazole ring with chloro substituents then led to the identification of trichloroindazole compound **173** with high inhibitory activity against PDAs (*k*_inact_/*K*_I_ values: PDA1, 20 ± 5; PAD2, 940 ± 240; PAD3, 5100 ± 400, 5,4000 ± 3000) (Figure 54). Furthermore, compound **173** inhibited PAD4-mediated H4 citrullination at low micromole concentrations. 

**Figure 55:**
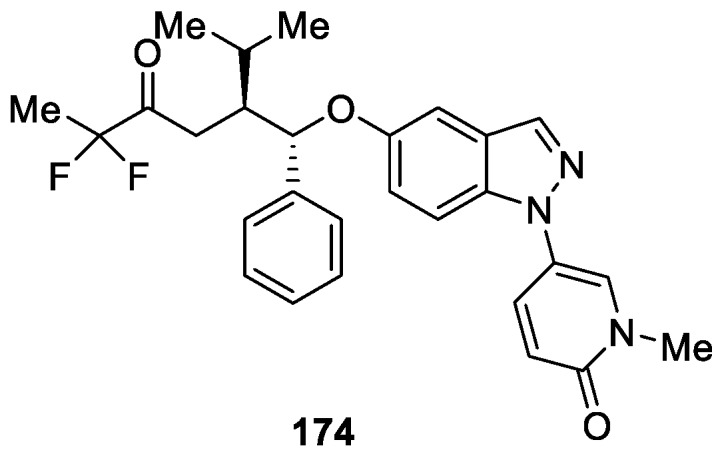
Chemical structure of 1*H*-indazole derivative **174**.

Ripa et al. [102] designed a novel series of 1*H*-indazole derivatives as potent glucocorticoid receptor (GR) modulators and conducted transactivation (TA) and transrepression (TR) assays. Based on the assays results, the compounds were further evaluated in functional assays measuring inhibition of lipopolysaccharide (LPS) induced tumor necrosis factor (TNF) α release in whole blood, upregulation of tyrosine aminotransferase (TAT) in primary hepatocytes, and downregulation of osteoprotegerin (OPG) in human fetal osteoblasts (hFOB). The experimental data ultimately lead to the discovery of compound **174**, which displayed excellent inhibitory activity against GR with IC_50_ value of 3.8 nM (Figure 55). In addition, compound **174** exhibited excellent efficacy in the streptococcal cell wall (SCW) reactivation model of joint inflammation.

### 3.5. Treatment of Parkinson’s Disease

**Figure 56:**
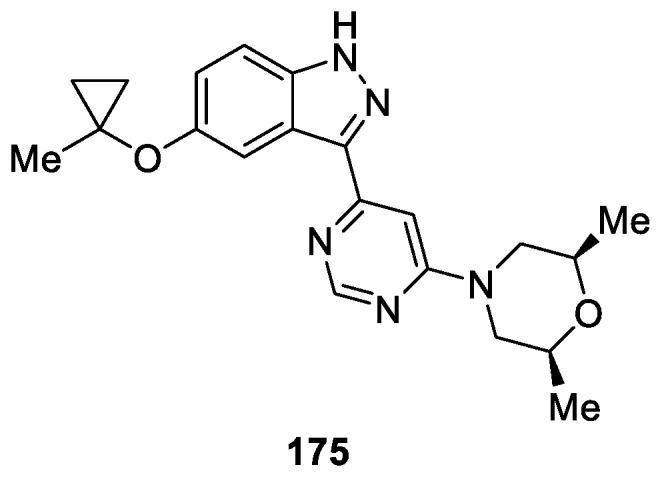
Chemical structure of 1*H*-indazole derivative **175** as LRRK2 inhibitor.

cis-2,6-Dimethyl-4-(6-(5-(1-methylcyclopropoxy)-1*H*-indazol-3-yl)pyrimidin-4-yl)morpholine (MLi-2) **178** is a structurally novel, highly potent drug-like compound developed by Merck [103,104] as a selective Leucine-Rich Repeat Kinase 2 (LRRK2) inhibitor for treatment of Parkinson′s disease (PD). MLi-2 has exhibited exceptional potency inhibitory activity against purified LRRK2 kinase with IC_50_ value of 0.76 nM in vitro. Additionally, in an evaluation performed in dephosphorylation of pSer935 LRRK2 and radioligand competition binding assays, compound **175** provided IC_50_ values of 1.4, 3.4 nM, respectively (Figure 56). Moreover, target engagement was confirmed through a dose-dependent reduction in the ratio of pS935 LRRK2 to total LRRK2 in the brains of rats after oral dosing with compound **175**. These data demonstrate the emergence of compound **175** as a key tool in the understanding of LRRK2 function and it possible suitability as a potent, highly selective, orally available compound to explore the LRRK2 inhibitor for treatment of Parkinson’s disease (PD).

**Figure 57:**
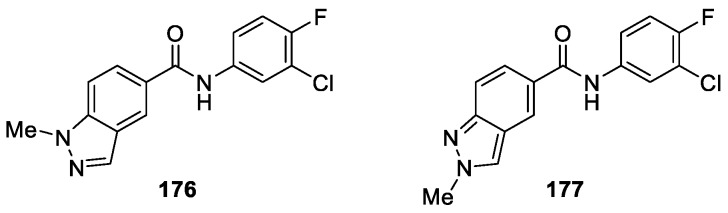
Chemical structures of *N*-alkylated indazole-5-carboxamide derivatives **176** and **177**.

Tzvetkov et al. [105] reported the synthesis of a class of *N*-alkyl-substituted indazole-5-carboxamide derivatives and optimized their Monoamine oxidases inhibitory activities to develop potential drug and radioligand candidates for the treatment of Parkinson’s disease (PD) and other neurological disorders. Some analogues showed promising inhibitory activities with nanomolar potency towards MAO-B and were moderately active against the MAO-A enzyme, respectively. The most promising derivatives among the tested compounds were **176** (IC_50_ hMAO-B 0.662 nM) and **177** (IC_50_ hMAO-B 8.08 nM, IC_50_ hMAO-A 0.56 μM) (Figure 57). The following studies confirmed that compounds **176** and **177** established binding interactions with hMAO-B via the carbonyl group of the carboxamide linkage and the N1 or N2 nitrogen of the indazole moiety.

### 3.6. Treatment of Anemia of Chronic Disease

**Figure 58:**
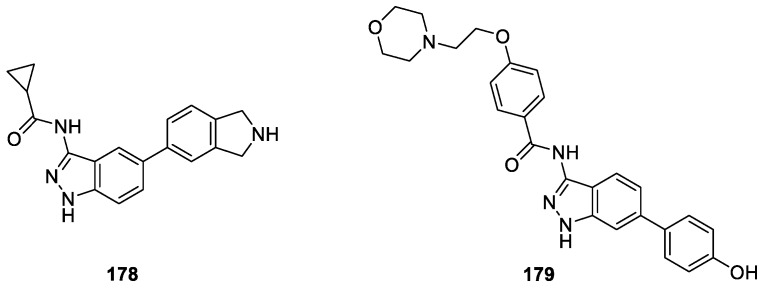
Chemical structures of 1*H*-indazole analogues **178** and **179**.

Fukuda et al. [106] reported a novel series of indazole derivatives as lead structures for potent hepcidin production inhibitors for treatment of anemia of chronic disease (ACD). The authors identified a 5-substituted indazole compound **178** through the screening of their compound library as a hepcidin production inhibitor, which showed weak activity in vitro (IC_50_ = 3.1 μM) (Figure 58). They adopted **178** as the leading compound and introduced a *para*-hydroxyphenyl group at the 6-position and [2-(morpholin-4-yl)ethoxy]benzamide group at the 3-position led to a potent hepcidin production inhibitor **179** (IC_50_ = 0.13 μM). Moreover, compound **179** exhibited serum hepcidin lowering effects in a mouse IL-6 induced acute inflammatory model.

**Figure 59:**
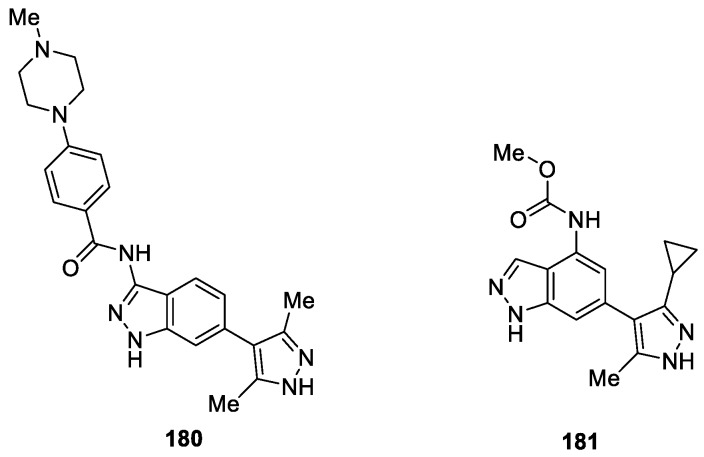
Chemical structures of 4,6-disubstituted indazole analogues **180** and **181**.

As a continuation of their research on the development of hepcidin production inhibitors for treating anemia of chronic disease (ACD), a series of new 4,6-disubstituted indazole compounds were designed, synthesized and evaluated as inhibitors of the above enzyme by Fukuda et al. [107]. Indazole derivative **180** was used as the leading compound to obtain a class of 4,6-disubstituted indazole analogues [108]. The optimization study revealed that compound **181** (DS28120313) appeared to be the most potent and bioavailable hepcidin production inhibitor with an IC_50_ value of 0.093 μM (Figure 59). Furthermore, compound **181** exhibited serum hepcidin-lowering effects in an interleukin-6-induced acute inflammatory mouse model.

### 3.7. Others Activitives

**Figure 60:**
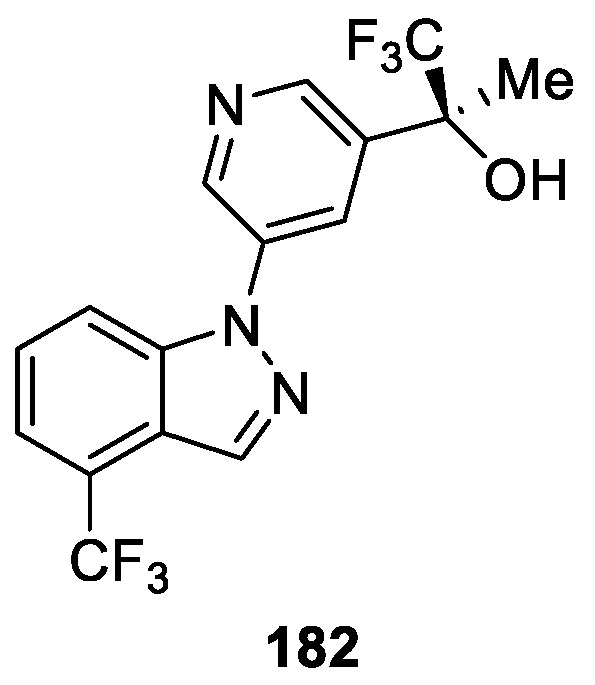
Chemical structure of 1*H*-indazole derivative **182**.

Hoyt et al. [109] reported the discovery and hit-to-lead optimization of a series of novel indazole derivatives with the aim of developing new CYP11B2 inhibitors for treating hypertension. Among the optimized compounds, **182** displayed high selectivity and the most potent inhibition of CYP11B2 with an IC_50_ value of 2.2 nM (Figure 60). It should also be mentioned that compound **182** showed lead-like physical and pharmacokinetic properties.

**Figure 61:**
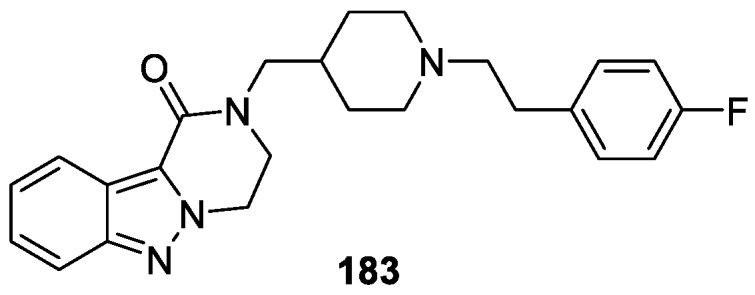
Chemical structure of 3,4-dihydropyrazino[1,2-*b*]indazol-1(2*H*)-one derivative **183**.

Furlotti et al. [110] reported the discovery and optimization of a series of novel 3,4-dihydropyrazino[1,2-*b*]indazol-1(2*H*)-one derivatives with the aim of developing new 5-hydroxytryptamine receptor 2A (5-HT_2A_) inhibitors for the treatment of hypertension. Among the optimized compounds, **183** displayed high selectivity and the most potent inhibition of 5-HT_2A_ with IC_50_ value of 18 nM (Figure 61). It should be mentioned that compound **183** showed clear ocular hypotensive action, superior in magnitude for the whole course of the experiment.

**Figure 62:**
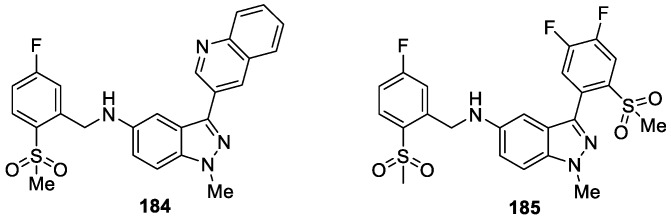
Chemical structures of 1*H*-indazole derivatives **184** and **185**.

Atobe et al. [111] reported a series of indazole derivatives as potent Sirt 1 activators by means of high-throughput screening. The design of the series was based on compound **184**, which bears 3-quinoly groups exhibiting potent Sirt 1 activating activity (EC_50_ = 1.49 μM) (Figure 62). Among these compounds, compound **185** appeared to show the best Sirt 1 activity (EC_50_ = 0.40 μM) and it also showed osteogenesis activity in a cell assay. The results confirmed that Sirt 1 activators were potential candidates for the treatment of osteoporosis.

**Figure 63:**
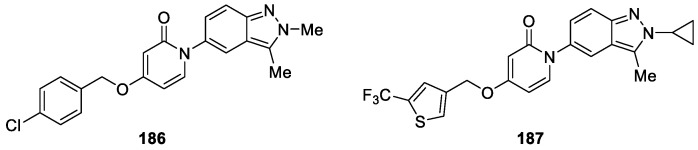
Chemical structures of 1-(2*H*-indazole-5-yl)pyridin-2(1*H*)-one derivatives **186** and **187**.

Igawa et al. [112] reported the synthesis of a novel series 1-(2*H*-indazole-5-yl)pyridin-2(1*H*)-one hybrids and investigated the potencies of the melanin-concentrating hormone receptor 1 (MCHR1). During the optimization, compound **186** showed binding affinity with MCHR1 (hMCHR1: IC_50_ = 35 nM) in the Ames test (Figure 63). Based on a putative intercalation of **186** with DNA, the authors introduced a cyclopropyl group on the indazole ring to decrease planarity, which led to the discovery of compound **187** without mutagenicity in TA1537. In particular, compound **187** exerted significant body weight reduction in diet-induced obese F344 rats and was expected to be a novel antiobesity agent based on MCHR1 antagonistic activity.

**Figure 64:**
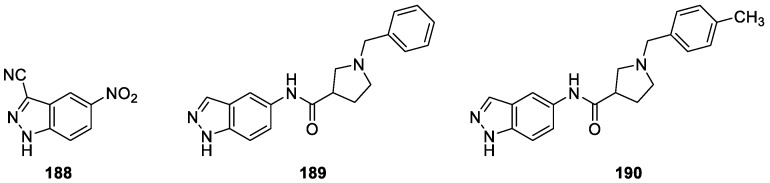
Chemical structures of N-substituted prolinamido indazole derivatives **188**, **189** and **190**.

Xie et al. [113] disclosed a series of new N-substituted prolinamido indazoles as potent Rho kinase (ROCK) inhibitors. The design of the series was based on the previously reported [114] compound 5-nitro-1*H*-indazole-3-carbonitrile **188**, which possess an IC_50_ value of 6.7 μM against ROCK I. The lead optimization, resulted in the discovery of the most promising analogues **189** (IC_50_ = 0.27 μM) and **190** (IC_50_ = 0.17 μM), among other active analogues (Figure 64). Moreover, compounds **189** and **190** displayed comparable vasorelaxant activity (vasorelaxant activity in rat aortic rings in both the high-potassium models with EC_50_ values of 4.00 and 3.47 μM, respectively; in norepinephrine models with EC_50_ values of 5.62 and 4.07 μM, respectively) to the approved drug fasudil (vasorelaxant activity in rat aortic rings in both the high-potassium and norepinephrine models with EC_50_ values of 5.47 and 4.65 μM, respectively) in rat aortic rings. The structure-activity relationship (SAR) demonstrated that the target compounds containing a β-proline moiety had improved activity against ROCK I relative to analogues bearing an α-proline moiety, and the target compounds bearing a benzyl substituent showed better inhibitory activity than those with a benzoyl substituent.

**Figure 65:**
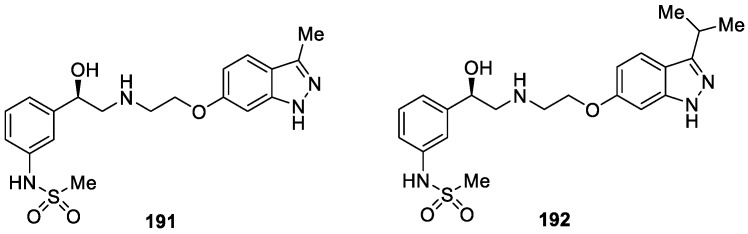
Chemical structures of 1*H*-indazole derivatives **191** and **192**.

Wada et al. [115] prepared a novel series of 1*H*-indazole derivatives and evaluated their biological activity and cardiovascular safety profile as human β_3_-adrenergic receptor (AR) agonists. Derivative **191** was identified as the initial hit compound, which exhibited significant β_3_-AR agonistic activity (EC_50_ = 21 nM), it also exhibited agonistic activity at the α1A-AR (EC_50_ = 219 nM, selectivity: α_1_A/β_3_ = 10-fold) (Figure 65). After systemic optimization, the authors developed the compound **192**, which possessed potent β_3_-AR agonistic activity (EC_50_ = 13 nM) and high selectivity (α_1_A/β_3_ = >769-fold). Compound **192** was also inactive towards β_1_ and β_2_-ARs and showed dose dependent β_3_-AR mediated relaxation of marmoset urinary bladder smooth muscle.

**Figure 66:**
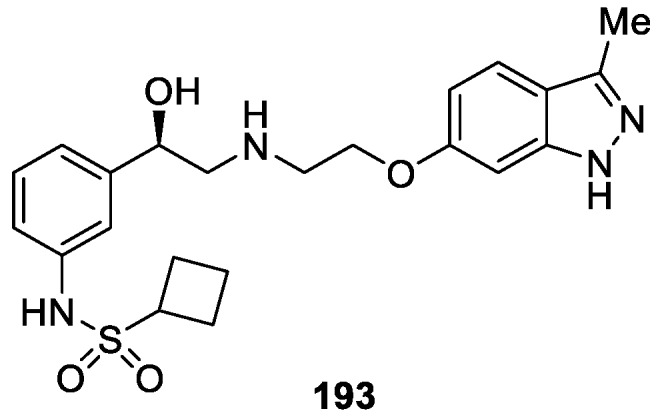
Chemical structure of 1*H*-indazole derivative **193**.

As a continuation of their research on the development of selective β_3_-adrenergic receptor (β_3_-AR) agonist, a series of new 1*H*-indazole compounds were designed, synthesized and evaluated as inhibitors of β_3_-AR agonist by Wada et al. [116]. Indazole derivative **192** was used as the leading compound to obtain a class of 1*H*-indazole analogues. The optimization study revealed that, compound **193** appeared to be the most potent β_3_-AR agonist with EC_50_ value of 18 nM (Figure 66). Additionally, compound **193** showed dose-dependent β_3_-AR-mediated responses in marmoset urinary bladder smooth muscle, had a desirable metabolic stability and pharmacokinetic profile, and did not obviously affect heart rate or mean blood pressure when administered intravenously (3 mg/kg) to anesthetized rats.

**Figure 67:**
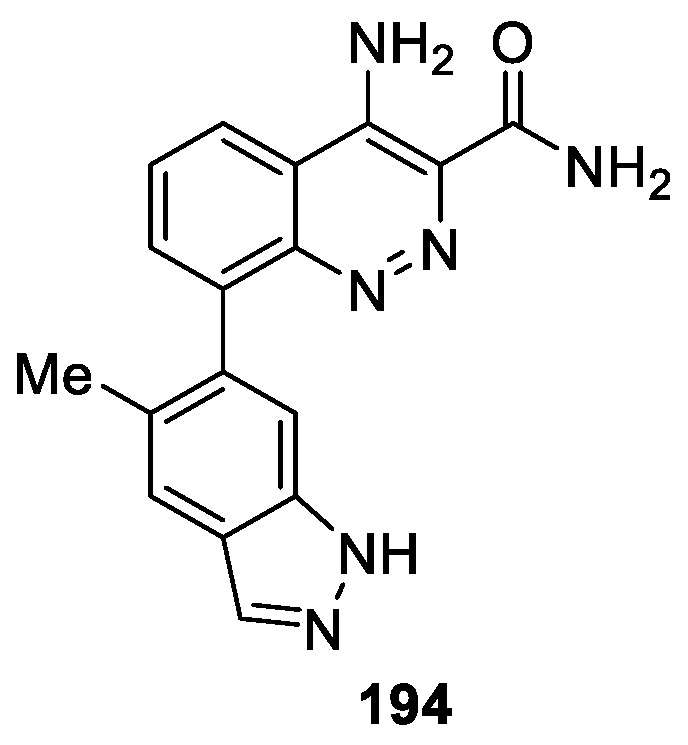
Chemical structure of 1*H*-indazole derivative **194**.

Smith et al. [117] reported a series of 4-aminocinnoline-3-carboxamide derivatives as Bruton’s tyrosine kinase (Btk) inhibitors based on a fragment-based screening approach. Compound **194** was identified through this process, which exhibited excellent inhibitory activity against Btk with IC_50_ value of 4.0 ± 0.3 nM (Figure 67). Moreover, compound **194** reduced paw swelling in a rat model of collagen induced arthritis.

**Figure 68:**
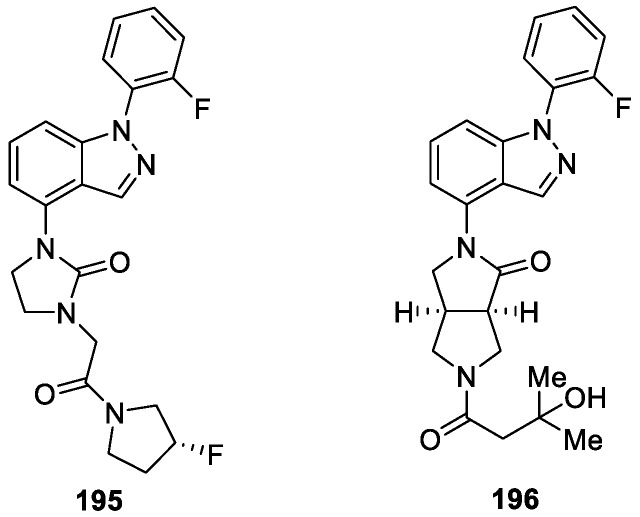
Chemical structures of 4-substituted-1*H*-indazole derivatives **195** and **196**.

Frost et al. [118] disclosed two series of indazole analogs that exhibited activity at Na_v_1.7. The 4-substituted indazole series exhibited good to moderate activity at Na_v_1.7. Furthermore, this series demonstrated improved pharmacokinetic profiles with good rat bioavailabilities and half-lives. Among them, (*S*)-3-fluoropyrrolidine **195** exhibited good activity at Na_v_1.7 (FRET IC_50_ = 0.37 μM, EP IC_50_ = 0.48 μM; manual EP IC_50_ = 0.57 μM), good selectivity versus Na_v_1.5 (>33 μM) and good pharmacokinetic properties (F = 83%; t_1/2_ = 1.7 h) (Figure 68). Compound **196** demonstrated moderate activity at Na_v_1.7 in the fluorescence resonance energy transfer (FRET) assay (FRET IC_50_ = 3.9 μM), excellent metabolic stability and good PK properties, but no activity at Na_v_1.5. In addition, compound **196** exhibited good activity in the acute rat mono-iodoacetate-induced osteoarthritis model at 10 and 30 mg/kg (EC_50_ = 2.5 μg/mL).

**Figure 69:**
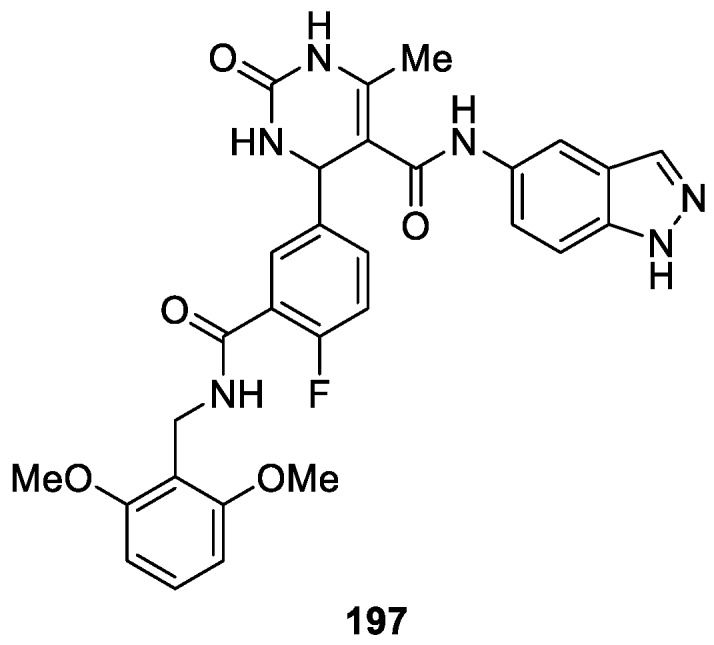
Chemical structure of 1*H*-indazole derivative **197**.

Utilizing a hybrid approach, Larsen et al. [119] prepared a novel series of 1*H*-indazole derivatives as potent and selective G protein-coupled receptor kinase 2 (GRK2) inhibitors. Among them, compound **197** was the most selective compound, which had an IC_50_ for GRK2 of 130 nM and no detectable inhibition of Rho-associated coiled-coil kinase 1 (ROCK1) (Figure 69). Co-crystal structures revealed that compound **197** binded snugly in the hydrophobic subsite of GRK2 with one methoxy group packing deep in the pocket.

**Figure 70:**
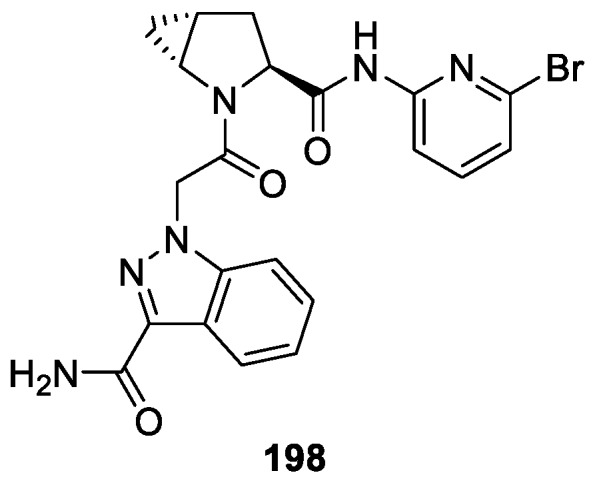
Chemical structure of 1*H*-indazole derivative **198**.

Lorthiois et al. [120] reported the synthesis of a novel series 1*H*-indazole hybrids and investigated their potencies of factor D (FD). During the optimization, compound **198** showed more potent inhibitory activity against FD with an IC_50_ value of 0.006 μM in the in vitro membrane attack complex (MAC) deposition assay (Figure 70). Moreover, compound **198** displayed an excellent oral pharmacokinetic (PK) profile in Sprague−Dawley rats and, following an oral dose (10 mg/kg) in Brown Norway rats, demonstrated a good distribution and sustained exposure in ocular tissues including the neural retina and the posterior eye cup (PEC), which comprises the sclera, retinal pigmented epithelium, and choroid.

**Figure 71:**
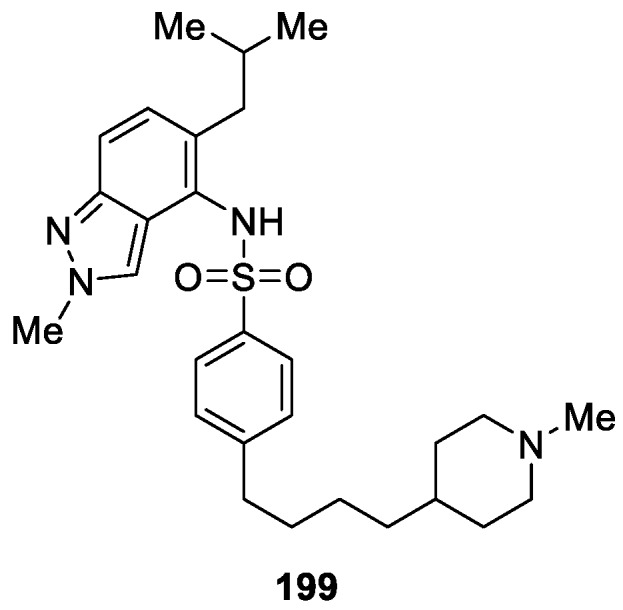
Chemical structure of 2*H*-indazole derivative **199**.

Read et al. [121] described the synthesis of a series of 2*H*-indazoles which were investigated for their trypanosoma brucei N-myristoyltransferase (NMT) inhibition efficiencies. The studies revealed that, among the synthesized and tested derivatives, **199** possessed the most potent inhibitory activity with IC_50_ values of 0.006 μM (Figure 71). In addition, compound **199** had good potency against the T. brucei parasite (EC_50_ = 5 nM), good microsomal stability (Cli = 2.9 mL/min/g), good selectivity (“S” = 47), good oral exposure, and good in vivo tolerability.

## 4. Conclusions

Indazole and its analogues are important scaffolds with a broad range of pharmacological activities. There has been an escalating interest in the development of compounds bearing indazole moiety against different kinds of diseases. Various bioactive moieties can easily be incorporated into indazole derivatives and a great amount of effort has been dedicated to the exploration of medicinal approaches for their preparation and evaluation of their biological activities. The present review not only updates recent developments in new reactions for the synthesis of indazole derivatives and their application in the medicinal field but also encourages medicinal chemists to further explore novel indazoles as potential drug candidates for useful therapeutics.

## Abbreviations

ACD	Anemia of chronic disease
ALK	Anaplastic lymphoma kinase
AR	Adrenergic receptor
Bcr-Abl	Break point cluster-Abelson
Btk	Bruton’s tyrosine kinasel
CD	Crohn’s disease
CDK	Cyclin-dependent kinase
CHK	Checkpoint kinase
DCE	1,2-Dichloroethane
DDQ	2,3-Dicyano-5,6-dichlorobenzoquinone
DFT	Density functional theory
DMPK	Drug metabolism and pharmacokinetics
DMPU	1,3-Dimethyl-3,4,5,6-tetrahydro-2(1H)-pyrimidinone
DMSO	Dimethyl sulfoxide
EGFR	Epidermal growth factor receptor
ER	Endoplasmic reticulum
ERK	Extracellular signal-regulated kinase
EZH	Enhancer of zeste homologue
FBLD	Fragment-based lead discovery
FDA	Food and drug administration
FGFR	Fibroblast growth factor receptor
FRET	Forster resonance energy transfer
FLT3	Fms-like tyrosine kinase 3
FtsZ	Filamentous temperature-sensitive protein Z
GK	Glucokinase
GPR	G-protein coupled receptor
GR	Glucocorticoid receptor
GRAs	Glucagon receptor antagonists
GRK	G protein-coupled receptor kinase
GSK3β	Glycogen synthase kinase 3 betas
hCAs	Human carbonic anhydrases
HDACs	Histone deacetylases
hEGR	Human ether-a-go-go related gene
hFOB	Human fetal osteoblasts
HFIP	Hexafluoroisopropanol
HT	Hydroxytryptamine
HTS	High-throughput screening
HUVEC	Human umbilical vein endothelial cells
IDO	Indoleamine-pyrrole 2,3-dioxygenase
IKKβ	Inhibitor of nuclear factor kappa-B kinase betan
JAK	Janus kinase
LE	Excellent ligand
LPS	Lipopolysaccharide
LRRK	Leucine-rich repeat kinase
MA	Molecular sieves
MAC	Membrane attack complex
MBA	Methoxybenzamide
MCHR	Melanin-concentrating hormone receptor
MIC	Minimum inhibitory concentration
NMT	N-myristoyltransferase
NMR	Nuclear magnetic resonance
NOD	Nucleotide-binding oligomerization domain-containing protein
NSCLC	Non-small cell lung cancer
OGTT	Oral glucose tolerance test
OPG	Osteoprotegerin
PAD	Protein arginine deiminase
PD	Pharmacodynamic
PDGFRβ	Platelet-derived growth factor receptor betan
PDK	Phosphoinositide-dependent kinase
PEC	Posterior eye cup
PI3Kδ	Phosphoinositide 3-kinase deltae
PIFA	[Bis-(trifluoroacetoxy)iodo]benzene
PK	Pharmacokinetic
PKC	Protein kinase C
PLK4	Polo-like kinase 4
RET	Rearranged during transfection
RIP	Receptor-interacting protein
ROCK	Rho-associated coiled-coil kinase
SAHA	Suberoylanilide hydroxamic acid
SAR	Structure-activity relationship
SCW	Streptococcal cell wall
SEDRs	Selective estrogen receptor degraders
SGRMs	Selective glucocorticoid receptor modulators
SNAr	Nucleophilic aromatic substitution
TA	Transactivation
TAT	Tyrosine aminotransferase
TGI	Tumor growth inhibition
TNFα	Tumor necrosis factor-alphas
TTK	Tyrosine threonine kinase
TR	Transrepression
TRPA	Transient receptor potential ankyrin-repeat
UC	Ulcerative colitis
ULK	Unc-51-like kinase
USP	Ubiquitin specific protease
VEGFR	Vascular endothelial growth factor receptor
